# Dietary Synbiotic Attenuated the Intestinal Inflammation in Weaned Piglets Challenged with *Escherichia coli* Lipopolysaccharide

**DOI:** 10.3390/ani15131832

**Published:** 2025-06-20

**Authors:** Gina-Cecilia Pistol, Valeria Cristina Bulgaru, Iulian Alexandru Grosu, Daniela Eliza Marin, Georgeta Ciurescu, Gheorghe Adrian Martău, Ionelia Taranu

**Affiliations:** 1 Laboratory of Animal Biology, INCDBNA-IBNA, National Research—Development Institute for Animal Biology and Nutrition, 077015 Balotesti, Romania; cristina.bulgaru@ibna.ro (V.C.B.); grosu.iulian@ibna.ro (I.A.G.); daniela.marin@ibna.ro (D.E.M.); ionelia.taranu@ibna.ro (I.T.); 2 Laboratory of Animal Nutrition and Biotechnologies, INCDBNA-IBNA, National Research—Development Institute for Animal Biology and Nutrition, 077015 Balotesti, Romania; ciurescu@ibna.ro; 3 Life Science Institute, University of Agricultural Sciences and Veterinary Medicine, 400372 Cluj-Napoca, Romania; adrian.martau@usamvcluj.ro

**Keywords:** synbiotic, piglets, weaning, intestinal inflammation, lipopolysaccharide

## Abstract

At weaning, piglets deal with numerous changes and stressors, which can lead to reduced feed intake, digestive disturbances, and gut inflammation. In this context, there is a compelling need to develop new and innovative nutritional strategies aimed at restoring intestinal balance in piglets after weaning and controlling the weaning-associated intestinal inflammation. This study investigated the effect of a diet, including a synbiotic additive (a mix of grape seed and camelina meals as the prebiotic and a lactobacilli mixture as the probiotic) on intestinal inflammation in piglets after weaning. An acute inflammation was induced by the intraperitoneally challenge with *Escherichia coli* lipopolysaccharide (LPS). The experimental groups were as follows: Control group (piglets fed a conventional corn-soybean meal-based diet), LPS group (piglets fed the Control diet and challenged with 80 µg/b.w. of LPS), SYN group (piglets fed a basal diet, including 5% prebiotic mix and 0.1% probiotic mix, SYN diet), and SYN+LPS group (piglets fed the SYN diet and challenged with 80 µg/b.w. of LPS). Using genomic and proteomic techniques, genes and proteins related to intestinal inflammation were measured in both the jejunum and colon. The results showed that the LPS challenge induced an exacerbated inflammatory response in the jejunum and colon of piglets, inducing an overexpression of a large palette of inflammation-related mediators, including lactate dehydrogenase (LDH) activity, cytokines (e.g., interleukin (IL)—IL-1β, IL-6, tumor necrosis factor alpha—TNF-α), and chemokines (e.g., monokine induced by interferon-gamma—MIG, regulated on activation, normal T cell expressed and secreted—RANTES). All these effects are prevented by the SYN diet, which controls the amplitude of intestinal inflammation induced by the LPS challenge in piglets. Overall, these results suggested that piglets, after weaning, fed the synbiotic diet are less susceptible to the LPS challenge. This diet might be used as a nutritional strategy to alleviate intestinal inflammation in piglets after weaning.

## Simple Summary

The weaning transition is associated with digestive disturbances and gut inflammation. That is why controlling weaning-associated intestinal inflammation in piglets using nutritional strategies is strongly needed. The purpose of this work was to assess the effects of a diet including a synbiotic additive (a mix of grape seed and camelina meals as prebiotic and a lactobacilli mixture as probiotics) on LPS-induced intestinal inflammation in piglets after weaning. The results showed that a synbiotic diet alleviated the inflammatory response in the jejunum and colon of piglets. These results suggested that the synbiotic diet might be used as a strategy to alleviate the enteric problems and intestinal inflammation in piglets after weaning. This study offers both practically and theoretical support to help raise healthier animals and can be applied in the livestock industry to improve intestinal health in piglets.

## 1. Introduction

One of the significant challenges in the pig industry is to ensure the health and growth of piglets, particularly during the weaning phase [1]. Weaning is a critical period involving stressors (environmental, nutritional, social), which can induce marked changes in gastrointestinal tract (GIT) physiology, microbiology, and immunology, leading to reduced food intake, digestive issues, gut inflammation, and limited nutrient absorption [1,2]. Also, weaning stress, especially the transition from maternal milk to a solid feed, activates the local (intestinal) immune responses and stimulate the production of pro-inflammatory cytokines, for example, interleukin (IL)-1β, IL-6, IL-8, tumor necrosis factor-α (TNF-α), and interferon-γ (IFN-γ) [3,4,5]. The overproduction of these cytokines leads to a change in intestinal structure, resulting in intestinal epithelial damage, inflammation, and barrier dysfunction [3,6].

These weaning-induced intestinal disturbances were attenuated for a long time by the use of the in-feed antibiotics. Also, this strategy was useful to promote the growth, general health status, and welfare of weaned piglets [7,8]. Accordingly, the long-term use of antibiotics led to the development of pathogen resistance with a negative impact on animal health, and their ban was implemented in 2006 (EC/2006). Therefore, there is a permanent need to find alternative solutions to the challenges faced by piglets during and after weaning that are effective for replacing antibiotics and for maintaining animal health status and well-being (e.g., innovative nutritional strategies). Of these, feed additives, supplements, probiotics, and bioactive compounds such as polyphenolic fibers, vitamins, and minerals from various sources have been intensively investigated in the last twenty years as replacements for antibiotics [7,9,10].

Studies in the field of probiotics have proven their capacity to improve gastrointestinal health [11]. It was reported that probiotics induced positive responses at the intestinal level, maintaining a normal immune response, modulating the release of cytokines [12], reducing the amplitude of local inflammation [13], and maintaining the epithelial integrity and the intestinal barrier function [14]. Similarly, studies with prebiotics, food, and feed ingredients that are rich in bioactive compounds such as polyphenols, fiber, vitamins, and minerals have demonstrated their anti-inflammatory, antioxidant, and growth-stimulating capacity of beneficial microorganisms in the gut [15,16].

The mixtures of prebiotics and probiotics, so-called synbiotics, could increase overall gut health by the synergistic action of both components [17], with more benefits than either the probiotic or prebiotic alone. Studies show that synbiotics can produce positive effects in two ways, either by improving animal health [18] or by promoting the growth of indigenous beneficial microflora (e.g., *Bifidobacteria*, *Lactobacilli*) [19]. For example, a synbiotic combination of xylanase and *Bacillus* sp. enhances jejunum villus height and reduces the pro-inflammatory cytokine TNF-α concentration and protein oxidation in the jejunum of post-weaning piglets [20]. Guevarra et al. demonstrated the positive modulation of the gut microbiota in weaned piglets challenged with *Escherichia coli* by a synbiotic combination of *Pediococcus acidilactici* and lactulose [21]. Although the number of studies is limited, the anti-bacterial effects of synbiotics in piglets demonstrate their potential to replace the in-feed antibiotics in the piglets’ diet. For example, a synbiotic combination of *Lactobacilli plantarum*, maltodextrin, and fructooligosaccharides reduced the adherence of *Escherichia coli* O8:K88 to the mucosa in the jejunum and colon of piglets [22]. A similar effect of a synbiotic containing flax-seed oil and *Lactobacillus plantarum* on the inhibition of pathogenic *Escherichia coli* growth and adherence to the jejunal and ileal mucosa was demonstrated in germ-free piglets [23]. In a human gut model, a synbiotic blend containing *Lactococcus lactis* and prebiotic fiber inhibited the growth of vancomycin-resistant *Enterococcus*, demonstrating the potential of this synbiotic in controlling antibiotic-resistant pathogens [24]. These studies highlighted the great potential for synbiotics to be used as antibiotic alternatives or to inhibit antibiotic-resistant pathogens.

Our previous studies demonstrated the beneficial effects of a synbiotic combination of grape pomace extract as a prebiotic and *Lactobacillus* sp. mixture as a probiotic on the intestinal inflammation in an in vitro model, leading to strong anti-inflammatory properties, due to the protective role of grape pomace compounds on the lactobacilli probiotic [25]. Also, the use of grape seed meal alone in enhancing the intestinal health of piglets proved its anti-inflammatory and anti-oxidant properties [26,27]. Given the current need to find a proper solution for a targeted outcome in weaning piglet nutrition, in the present study, we aimed to investigate the in vivo effects of a diet including a synbiotic additive (a mix of grape seed and camelina meals as the prebiotic and a lactobacilli mixture as the probiotic) on intestinal inflammation in piglets after weaning. Genomic and proteomic techniques were used to evaluate the effects of a diet containing a synbiotic mix in post-weaned piglets challenged with *Escherichia coli* LPS on the expression of genes and proteins involved in the inflammatory response in the jejunum and colon of piglets, as major sites affected by *Escherichia coli* recurring infections in piglets after weaning.

## 2. Materials and Methods

### 2.1. Synbiotic Diet Components Preparation and Characterization

#### Prebiotic Mix Preparation and Determination of Composition in Bioactive Compounds

Grape seed and camelina meals were purchased from local providers (S.C. OLEOMET S.R.L., Giurgiu, Romania and Savoarea Soarelui, Oradea, Romania) as dried materials. After the evaluation of chemical basic composition, a mix of grape seed meal and camelina meal (3:1 ratio) was prepared and included in the complete feed. The ratio of 3:1 grape seed meal/camelina meal was selected based on previous analyses on a panel of mixes containing different rates of inclusion of grape seed/camelina meals, showing that this ratio of 3:1 (grape seed/camelina meals) had a high content of bioactive compounds (polyphenols). Bioactive compounds concentration of the prebiotic mix was determined using the following methods/polyphenol by the Folin–Ciocalteu method, as described by [25]; the fatty acid by the gas chromatography method, as described by [28]; microminerals Copper (Cu), Iron (Fe), Zinc (Zn), and Manganese (Mn) by atomic absorption spectrometry (SOLAAR M6 Dual Zeeman Comfort, Cambridge, UK) after microwave digestion and mineralization with nitric acid (EC/152/2009 STAS 9597/16-86STAS 9597/17-86); carbohydrates (fructose, glucose, sucrose, maltose) separated on Kromasil-NH2 column and detected by HPLC, as described by [29]; and organic acids (oxalic, citric, tartric, succinic, malic, and fumaric) separated on a CarboSep Coregel 87H3 Column and detected by HPLC, as described by [29].

Prebiotic mix from SYN diet had a total polyphenol content of 3267.9 mg GAE/100 g sample (Table 1(A)) and a high antioxidant activity (DPPH) (52.45%). Among the polyphenols, the prebiotic mix was rich in flavonoids (catechins, caffeic acid, gallic acid, vanilic acid, epicatechin Table 1(A)). The prebiotic mix also has a high content of polyunsaturated fatty acids (PUFAs), especially ω-6 PUFAs, with an increased concentration of cis-linoleic acid (47.71 mg/100 g, Table 1(B)). The analysis of micromineral and fiber content showed a high content of Cu, Fe, Mn, Zn, and fiber (Table 1(C)) as well as a rich level of carbohydrates, such as sucrose, glucose, and fructose, and also of organic acids (e.g., succinic acid, tartric acid, and oxalic acid, Table 1(C)).

### 2.2. Probiotic Mix Preparation

*Lactobacillus acidophilus* (ID 11692), *Lactobacillus paracasei* (ID 13239), and *Lactobacillus rhamnosus* (ID IBNA02) strains were used in this study. After obtaining bacterial biomass through 18 h of aerobic fermentation in BIOSTAT MD bioreactor (B. Braun Biotech International GmbH, Melsungen, Germany), each lactobacillus strain was microencapsulated by spray drying using Niro Mobile Minor (GEA, Düsseldorf, Germany) and maltodextrin- D-glucose- casein as matrix.

Lactobacilli mix was prepared as a ratio of *Lactobacillus acidophilus*, *Lactobacillus paracasei*, and *Lactobacillus rhamnosus,* being 1:1:1, according to the lactobacilli mix used in our previous in vitro study [25]. A total of 1.5 × 10^7^ CFU/g of lactobacilli mix was thoroughly mixed with the feed, the lactobacilli inclusion rate (0.1%) being established based on reported literature data [30].

### 2.3. Animals and Treatments

Thirty-two crossbred, 21-day-old piglets (TOPIGS-40) after weaning, with an average initial body weight of 7.5 ± 0.2 kg, were randomly assigned to four experimental groups: group 1 (piglets fed Control diet), group 2 (piglets fed Control diet and challenged with 80 µg/b.w. of LPS), group 3 (piglets fed SYN diet), and group 4 (piglets fed SYN diet and challenged with 80 µg/b.w. of LPS). The experiment started immediately after the piglets were weaned and lasted 21 days. Piglets from each experimental group had ad libitum access to water and feed during the experimental period. The animals were housed in pens (4 piglets/group/pen, two pens/experimental group) within the experimental base of the National Research—Development Institute for Animal Biology and Nutrition, Balotesti, Romania.

Two diets, a Control diet (complete feed—corn-soybean based diet) and a SYN diet (basal diet with 5% prebiotic mix and 0.1% probiotic mix included), were used in this study. Both Control and SYN diets are designed to respect the weaning piglets’ nutritional requirements (National Research Council—NRC 2012, [31]). At the beginning of the experiment, feed samples were collected, and basic and chemical composition (crude protein, crude fat, crude fibers, and ash) of experimental diets were evaluated using the Weende technique, according to the International Organization for Standardization (ISO) methods (SR EN ISO, 2010, [26]). The composition of diets used in this study is presented in Table 2.

Ethics Statements: The experimental protocol was approved by the Ethical Committee (no. 4406/2023) of INCDBNA Balotesti, and the animal handling was performed following rules for handling and protection of animals used for experimental purposes with EU Council Directive 98/58/EC and Romanian Law 43/2014 and in compliance with the ARRIVE guidelines.

On day 21 of the experiment, piglets in groups 2 (LPS) and 4 (SYN + LPS) were intraperitoneally challenged with sterile saline solution containing 80 µg/b.w. LPS to induce intestinal inflammation as described in [32], whereas pigs in groups 1 (Control) and 3 (SYN) were administered an equal volume of sterile saline solution. For a more effective induction of the inflammation symptoms, LPS solution was used, containing LPS isolated from *Escherichia coli* O26: B6 strain (Sigma Aldrich, St. Louis, MO, USA), from *Escherichia coli* O111: B4 strain (Sigma Aldrich, St. Louis, MO, USA), and from *Salmonella enterica serotype enteritidis* strain (Sigma Aldrich, St. Louis, MO, USA) in a ratio of 3:1:1. At 3 h post-challenge, blood was collected from all piglets. After that, piglets were slaughtered by electric stunning according to the EU Council Directive 2010/63/CE. During both LPS challenge and slaughtering, efforts have been made to minimize animal suffering and stress. Samples of the jejunum and colon were collected on ice and stored at −80 °C until the analyses.

### 2.4. Determination of LPS Toxicity in Plasma, Jejunum, and Colon Through Lactate Dehydrogenase (LDH) Assay

The LDH activity in plasma, jejunum, and colon was quantified using the Lactate Dehydrogenase Activity Assay Kit (Sigma Aldrich, St. Louis, MO, USA) according to the manufacturer’s protocol. Briefly, 25 µL of plasma or jejunum/colon lysates was mixed with 25 µL of assay buffer in a 96-well plate. An amount of 50 µL of master reaction mix (substrate mix diluted in the assay buffer) was added to each well. After 2–3 min, the initial measurement of the absorbance at 450 nm was performed. The plate was further incubated at 37 °C, taking measurements every 5 min. Absorbance measurements were taken every 5 min, and values within the linear range (up to 12.5 nmol/well) were used for calculating enzyme activity. Samples exceeding this range were diluted and reanalyzed. The final measurements obtained after 5 min of incubation were in the linear range and were selected for calculating the enzyme activity. The results were expressed as mU/mL (for plasma samples) and as mU/µg protein (for jejunum and colon). The protein concentration in jejunum and colon samples was determined using the BCA protein assay kit according to the manufacturer’s recommendation.

### 2.5. Quantitative PCR (qPCR) Array Analysis

Total RNA was extracted from jejunum and colon samples using RNeasy Plus Universal Mini Kit (QIAGEN GmbH, Hilden, Germany) according to the manufacturer’s recommendations. The complementary DNA (cDNA) was generated from the total RNA using the M-MuLV reverse transcriptase kit (Thermo Fisher Scientific, Waltham, MA, USA) according to the manufacturer’s protocol.

ExProfile™ Gene qPCR Array (GeneCopoeia, Rockville, MD, USA), a customized 96-well plate array, was used to study the gene expression of 40 key genes involved in the intestinal inflammatory response (Supplementary Table S1). In each well, a mixture of 25 ng cDNA template containing lyophilized primers was added, plus 10 µL SYBR Green qPCR Master Mix (Promega, Madison, WI, USA) and nuclease-free water (to a final volume of 20 µL), according to the manufacturer’s protocol. Real-time PCR was performed by using the cycling protocol described by [26]. The threshold cycle (Ct) value for each gene was determined as a relative value by the normalization against two reference genes (*ACTB* and *GAPDH*), selected based on their high stability from a panel of five housekeeping genes. The improved normalization of the results was based on this selection and was a measure of their constant expression in all analyzed samples, as described by [25]. Results were expressed as relative fold change (Fc) compared to the Control group.

### 2.6. Detection of Cytokine Protein Expression by Protein Array Analysis

Porcine Cytokine Array C1 kit (Ray Biotech, Peachtree Corners, GA, USA) was used to detect the relative expression levels of cytokines in the jejunum and colon according to the manufacturer’s protocol. The detection of signals was performed using a chemiluminescent reagent and ChemiDoc imaging system (Bio-Rad, Hercules, CA, USA). ImageJ software (https://imagej.net/ij/ (accessed on 15 July 2024)) was used for quantification. The forty-eight markers were tested in duplicate and are listed in Supplementary Table S2.

### 2.7. Enzyme-Linked Immunosorbent Assay (ELISA)

Jejunum and colon from piglets were analyzed by ELISA for quantification of the protein concentration of IL-1β, IL-6, IL-8, and IFN-γ pro-inflammatory cytokines and of anti-inflammatory cytokine IL-10 using specific pig ELISA kits (R&D Systems, Minneapolis, MN, USA) according to the manufacturers’ instructions, as described by [33].

### 2.8. Statistical Analysis

Results are presented as mean ± SEM. One-way ANOVA tests, followed by Fisher’s procedure of least square differences, were used for the comparison between experimental groups (StatView software 6.0 SAS Institute, Cary, NC, USA). To evaluate the overall treatment effect, the model effects were diet (without or with SYN), LPS challenge (yes or no), and their interaction using two-way mixed ANOVA test (StatView software 6.0—SAS Institute, Cary, NC, USA), and statistical significance was considered at *p* < 0.05. Pettitt’s and Buishand’s tests were used to assess the homogeneity of the results (GraphPad Prism 9.3.0. software, Boston, MA, USA). The results were tested for the normal distribution of the data using the NORM.DIST function of MS-Excel. The cluster analysis of experimental groups was performed using principal component analysis (PCA) multivariate method and heatmaps (GraphPad Prism 9.3.0. software and ClustVis web tool [34], respectively).

## 3. Results

### 3.1. Effects of SYN Diet on LPS-Induced Damage

No statistical differences were observed in terms of animal performances: final body weight, average daily gain (ADG), average daily feed intake (ADFI), and feed conversion ratio (FCR).

#### 3.1.1. Effects on LDH Activity in Plasma

**Figure 1:**
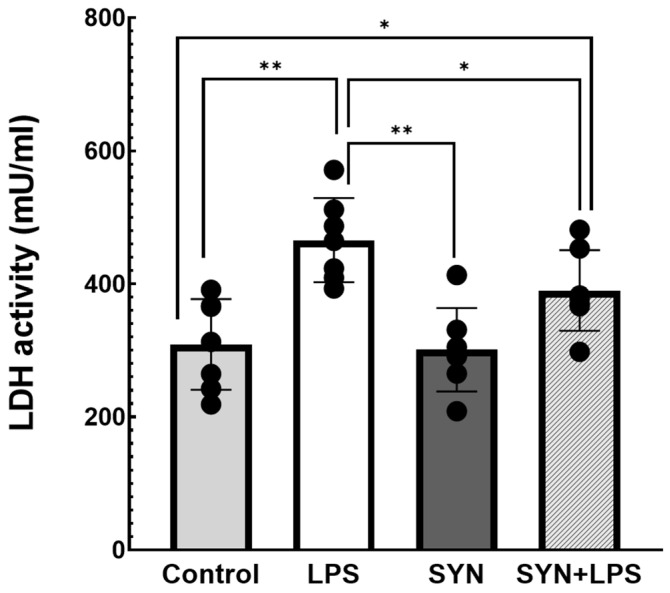
**The effects of the SYN diet on LPS-induced LDH activity in plasma.** Post-weaned piglets were fed for 21 days with a Control diet (Control and LPS groups) or SYN diet (SYN and SYN+LPS groups). At the end of the feeding trial, piglets from the LPS and SYN+LPS groups were challenged with 80 µg/b.w. LPS. After 3h, blood samples from all animals (n = 8) were collected, and the obtained plasma was used for quantification of LDH activity, as described in the Materials and Methods section. The results are presented as mean ± standard errors. A two-way ANOVA followed by Fisher’s test was used to analyze the effect of experimental treatments on LDH activity. *, ** Groups with unlike symbols were significantly different (*p* < 0.05 for *; *p* < 0.005 for **).

Given the fact that an increased LDH activity in blood is considered a marker of intestinal inflammation [35], we measured the activity of LDH in plasma collected from post-weaned piglets fed the Control diets, SYN diets, and SYN diets challenged or not challenged with LPS. Our results, presented in Figure 1, showed that the LPS challenge induced a significant increase in LDH activity in plasma when compared to the Control group (465.6 ± 22.3 mU/mL activity in the LPS group vs. 308.8 ± 24.1 in the Control group, *p* < 0.001, Figure 1). The SYN diet prevented the increase in LDH activity induced by the LPS treatment (390.0 ± 21.3 mU/mL, *p* = 0.041 vs. LPS group, Figure 1), while the SYN diet alone had no effect on LDH activity compared to the Control group (Figure 1).

#### 3.1.2. Effects on LDH Activity in the Jejunum and Colon

**Figure 2:**
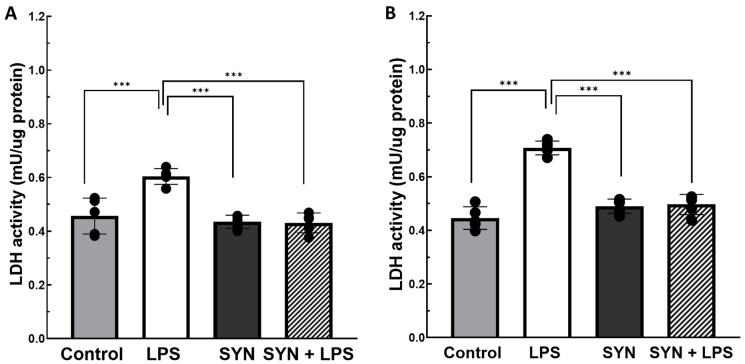
**The effects of the SYN diet on LPS-induced LDH activity in the jejunum (A) and colon (B).** Post-weaned piglets were fed for 21 days with a Control diet (Control and LPS groups) or SYN diet (SYN and SYN+LPS groups). At the end of the feeding trial, piglets from the LPS and SYN+LPS groups were challenged with 80 µg/b.w. LPS. After 3 h, jejunum and colon samples from all animals (n = 8) were collected and used for quantification of LDH activity, as described in the Materials and Methods section. The results are presented as mean ± standard errors. A two-way ANOVA followed by Fisher’s test was used to analyze the effect of experimental treatments on LDH activity. *** Groups with unlike symbols were significantly different (*p* < 0.001 for ***).

Next, we investigated the effects of the experimental diets and LPS treatment on LDH activity in the jejunum and colon of post-weaned piglets. The challenge of piglets with LPS significantly increased the LDH activity in both the jejunum and colon compared to untreated (Control) piglets (in jejunum: 0.60 ± 0.01 mU/µg protein in the LPS group vs. 0.46 ± 0.02 mU/µg protein in the Control group, *p* < 0.001, Figure 2A; in colon: 0.71 ± 0.01 mU/µg protein in the LPS group vs. 0.45 ± 0.01 mU/µg protein in the Control group, *p* < 0.001, Figure 2B).

Similarly with LDH activity in plasma, the SYN diet counteracted the effect of LPS in the jejunum and colon (0.44 ± 0.01 mU/µg protein, *p* < 0.001 vs. LPS group in the jejunum and 0.49 ± 0.01 mU/µg protein, *p* < 0.001 vs. LPS group in the colon, Figure 2A,B). Again, the SYN diet alone does not affect intestinal LDH activity compared to the Control group (Figure 2A,B).

### 3.2. Effects of SYN Diet on LPS-Induced Inflammation in the Jejunum

For a global overview of the effects of the experimental diets and LPS challenge on intestinal inflammation, qPCR and protein arrays were performed in both the jejunum and colon. The mediators of inflammation were grouped based on their pro-inflammatory/anti-inflammatory roles, and the results are presented below.

#### 3.2.1. Effects on Inflammation-Related Gene Expression in Piglets’ Jejunum

**Figure 3:**
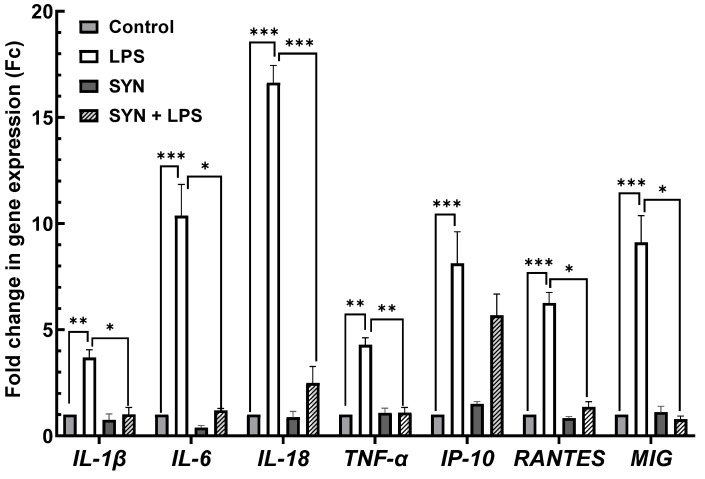
**The effects of the LPS and SYN diet on the genes coding for selected inflammatory markers in the jejunum** by qPCR array as described in the Materials and Methods section. The results showing *IL-1β*, *IL-6*, *IL-18*, *TNF-α*, *IP-10*, *RANTES,* and *MIG* gene expression are presented as mean ± standard errors. A two-way ANOVA followed by Fisher’s test was used to analyze the effect of experimental treatments on gene expression. Values of *p* < 0.050 were considered significant. *, **, *** Groups with unlike symbols were significantly different (*p* < 0.050 for *, *p* < 0.010 for **, *p* < 0.001 for ***).

The results of the qPCR array analysis presented in Supplementary Table S3 demonstrated the induction of an inflammatory response in the jejunum by the LPS challenge, more than 83% (30/36) of the analyzed genes coding for pro-inflammatory mediators being up-regulated in LPS-treated piglets. Of these, in Figure 3, we presented the most significant up-regulated genes, coding for pro-inflammatory regulator *IL-18* (16.6-fold increase, *p* < 0.001), for the master pro-inflammatory cytokine *IL-6* (10.4-fold increase, *p* < 0.001), and for interferon (IFN)-γ—inducible chemokines *MIG* (9.1-fold increase, *p* < 0.001) and *IP10* (8.1-fold increase, *p* < 0.001)—compared to the Control group (Figure 3, Supplementary Table S3).

By contrast, in the jejunum of piglets fed the SYN diet, 50.0% of pro-inflammatory cytokine genes remained unmodified compared to the Control group (Supplementary Table S3). In the case of a total of 83.3% (25/30) of increased pro-inflammatory mediator genes affected by the LPS treatment, the SYN diet prevented their up-regulation (in the SYN+LPS group). In the case of the SYN+LPS group, the SYN diet can ameliorate the LPS-induced inflammation; 80% (20/25) of the genes had expression levels appropriate from those found in the Control (pro-inflammatory cytokines *IL-6*: -9.17-fold reduction, *p* = 0.025 vs. LPS group; *TNF-α*: -3.19-fold reduction, *p* = 0.031 vs. LPS group; *IL-1β*: -2.68-fold reduction, *p* = 0.038 vs. LPS group, chemokines *MIG* -8.32-fold reduction, *p* = 0.020 vs. LPS group and *RANTES* -4,9-fold reduction, *p* = 0.018 vs. LPS group, Figure 3, Supplementary Table S3). The SYN and SYN+LPS groups had no significant effect on pro-inflammatory cytokine genes when compared to the Control group (Figure 3, Supplementary Table S3).

In the case of genes coding for anti-inflammatory mediators, the SYN diet and LPS challenge did not affect the expression of these genes in the jejunum (Supplementary Table S3).

**Figure 4:**
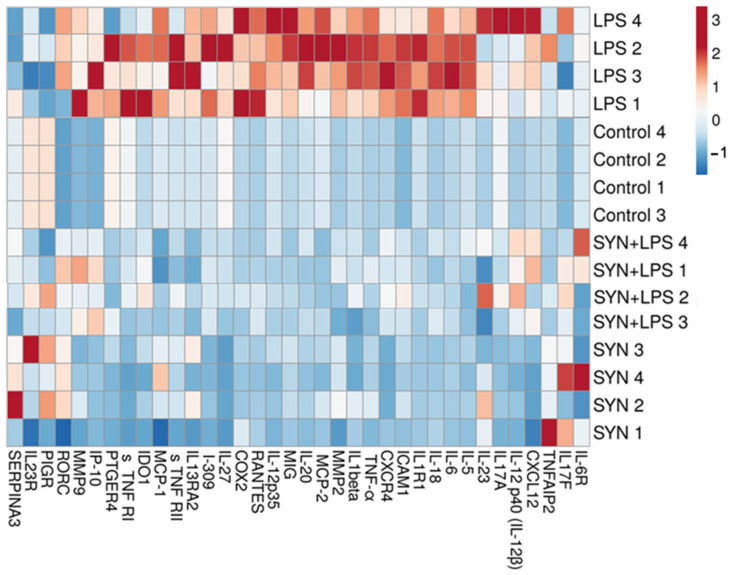
**The effects of the diets and LPS challenge on pro-inflammatory-related genes in the jejunum**. The heatmap represents the relative expression levels of genes at the end of the feeding experiment and after 3 h of the LPS (LPS and SYN+LPS groups) or saline challenge (SYN group) compared to the unchallenged group (Control group). The magnitude of the gene expression level is represented by a color scale (top) going from low (blue) to high (red).

The heatmap showed a distinct difference between the LPS-challenged group and all other experimental groups (Figure 4). Positive correlations between the LPS-treated piglets and the expression of genes coding for *IL-6*, *IL-18*, *IL-1β*, *TNF-α*, *RANTES,* and *MIG* were found (Figure 4). By contrast, both the SYN and SYN+LPS diets registered negative correlations with pro-inflammatory genes (Figure 4).

#### 3.2.2. Effects on Inflammation-Related Protein Expression in Piglets’ Jejunum

**Figure 5:**
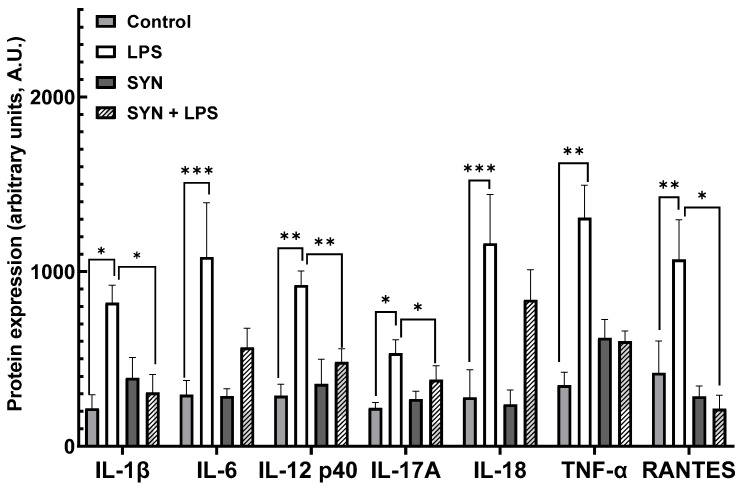
**The effects of the LPS and SYN diet on the selected inflammatory proteins in the jejunum.** The results showing IL-1β, IL-6, IL-12 p40, IL-17A, IL-18, TNF-α, and RANTES protein expression are presented as mean ± standard errors. A two-way ANOVA followed by Fisher’s test was used to analyze the effect of experimental treatments on gene expression. Values of *p* < 0.050 were considered significant. *, **, *** Groups with unlike symbols were significantly different (*p* < 0.050 for *; *p* < 0.010 for **, *p* < 0.001 for ***).

Further, we analyzed the effect of the LPS and SYN diet on the expression of the inflammatory proteins using the protein array technique. The results presented in Supplementary Table S4 showed that after 3 h of the LPS treatment, an increase of 89% (32/36) in pro-inflammatory proteins was registered in the jejunum of piglets. Similarly with the gene analysis, the main pro-inflammatory proteins were significantly affected by the LPS challenge (IL18: 4.0-fold increase, *p* < 0.001 vs. Control; IL-6: 3.7-fold increase, *p* < 0.001 vs. Control, RANTES 2.5-fold increase, *p* = 0.0008 vs. Control, Figure 5, Supplementary Table S4). Seventy-eight percent of pro-inflammatory-related proteins were not modified by the SYN diet alone in the jejunum (Supplementary Table S4), while in the SYN+LPS group, SYN had protective effects against the LPS-induced inflammatory proteins over-expression. Briefly, the SYN diet ameliorated the up-regulation of 69% (22/36) of LPS-affected proteins (IL-12p40: -1.6-fold reduction, *p* = 0.013 vs. LPS, RANTES: -2.0-fold reduction, *p* = 0.008 vs. LPS, IL-17A: -1.9-fold reduction, *p* = 0.042 vs. LPS, Figure 5, Supplementary Table S4), and 55% (12/22) of them remained unmodified when compared to the Control group. The LPS challenge affected the protein expression for TGF-α and TGF-β1 anti-inflammatory mediators (1.8-fold increase, *p* = 0.050 vs. Control and 2.1-fold increase, *p* = 0.050 vs. Control, respectively, Supplementary Table S4), while the rest of the anti-inflammatory proteins remained unaffected. The SYN diet did not alleviate the increase in anti-inflammatory TGF-α and TGF-β1 markers caused by LPS action (Supplementary Table S4).

**Figure 6:**
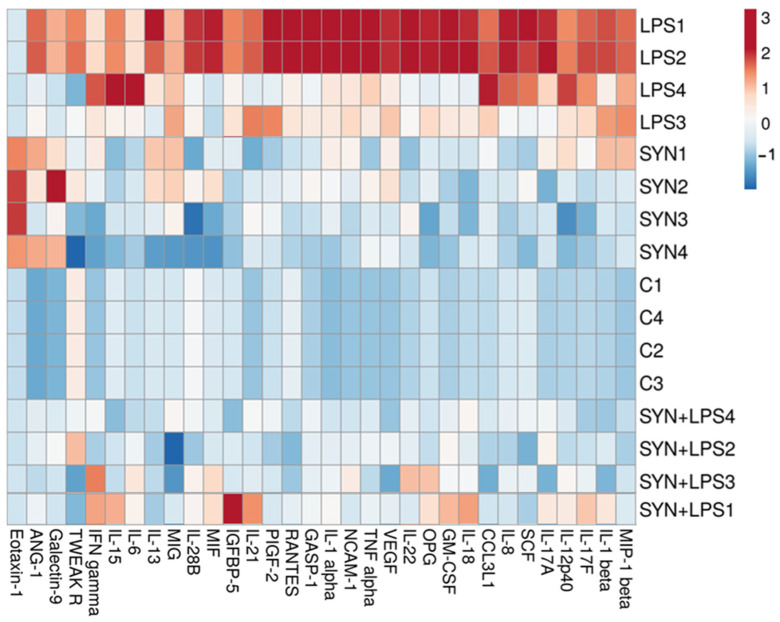
**The effects of the diets and LPS challenge on inflammatory-related proteins in the jejunum**. The heatmap represents the relative expression levels of genes at the end of the feeding experiment and after 3h of the LPS (LPS and SYN+LPS groups) or saline challenge (SYN group) compared to the unchallenged group (Control group). The magnitude of the protein expression level is represented by a color scale (top), going from low (blue) to high (red).

The heatmap from Figure 6 also showed the changes in the expression levels of pro-inflammatory proteins within experimental groups. Again, the LPS group was a distinct group, positively associated with the expression of pro-inflammatory proteins, while both the SYN and SYN+LPS groups were associated mainly with mild effects or negative effects on the expression of inflammatory mediators (Figure 6).

### 3.3. Effects of SYN Diet on LPS-Induced Inflammation in the Colon

#### 3.3.1. Effects on Inflammation-Related Gene Expression in Piglets’ Colon

**Figure 7:**
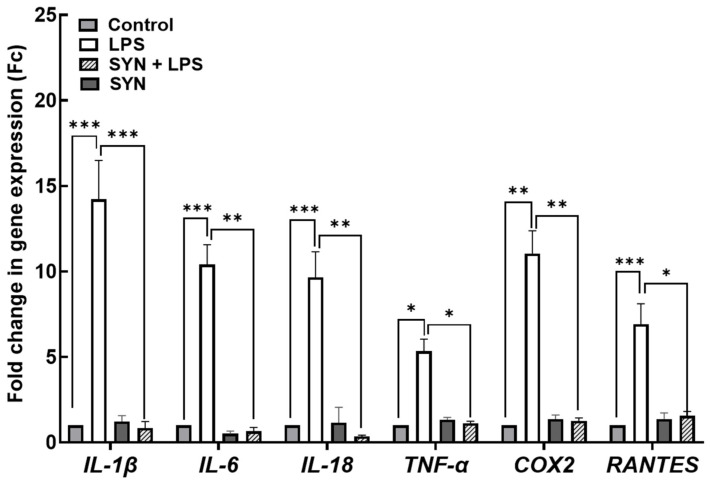
**The effects of the LPS and SYN diet on the genes coding for selected inflammatory markers in the colon**. The results showing *IL-1β*, *IL-6*, *IL-18*, *TNF-α*, *COX2*, and *RANTES* gene expression are presented as mean ± standard errors. A two-way ANOVA followed by Fisher’s test was used to analyze the effect of experimental treatments on gene expression. Values of *p* < 0.050 were considered significant. *, **, *** Groups with unlike symbols were significantly different (*p* < 0.050 for *; *p* < 0.010 for **, *p* < 0.001 for ***).

Using the qPCR array, we found that inflammation-related genes were up-regulated in the colon after the LPS challenge compared with the Control piglets (Supplementary Table S5). A total of 60% (21/36) of genes coding for pro-inflammatory markers were up-regulated in the colon of LPS-treated piglets, the most affected genes being *IL-1β* (14.2-fold increase, *p* < 0.001 vs. Control), *IL-6* (10.4-fold increase, *p* < 0.001 vs. Control), *IL-18* (9.6-fold increase, *p* < 0.001 vs. Control), *COX 2* (11.0-fold increase, *p* = 0.005 vs. Control), *RANTES* (6.9-fold increase, *p* = 0.0008 *vs.* Control), and *TNF-α* (5.3-fold increase, *p* = 0.008 *vs.* Control) (Figure 7, Supplementary Table S5). The SYN diet alone does not affect 67% (24/36) of the inflammatory-related genes in the colon compared to the Control diet (Supplementary Table S5). However, the SYN diet protected the expression of 90% (19/21) of the total genes affected by the LPS challenge, maintaining 76% of them (16/21) at the level of the Control samples.

The expression of genes coding for *TIMP-1* and *TIMP-2* anti-inflammatory mediators was significantly reduced after the LPS challenge in the piglets’ colon (Supplementary Table S5), whereas the SYN diet prevented the decrease in *TIMP-1* mRNA (Supplementary Table S5).

**Figure 8:**
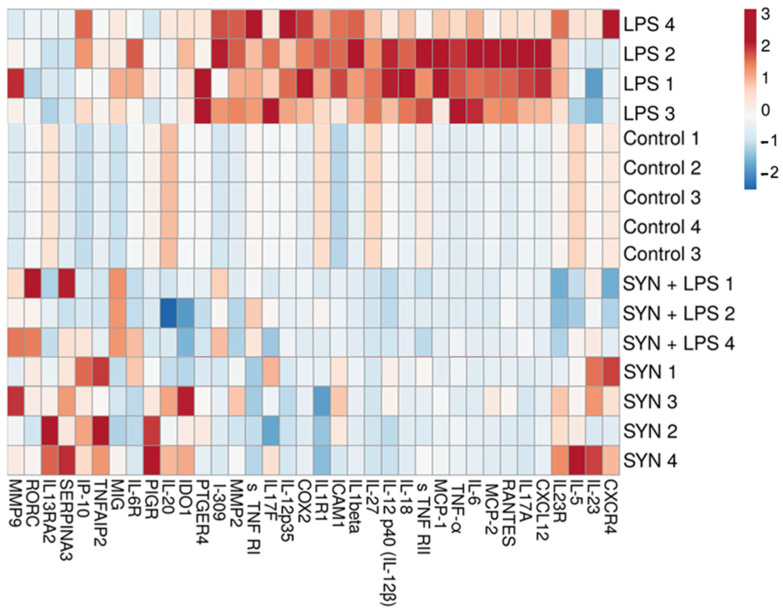
**The effects of the diets and LPS challenge on inflammatory-related genes in the colon**. The heatmap represents the relative expression levels of genes at the end of the feeding experiment and after 3 h of the LPS (LPS and SYN+LPS groups) or saline challenge (SYN group) compared to the unchallenged group (Control group). The magnitude of the gene expression level is represented by a color scale (top), going from low (blue) to high (red).

To investigate the relationship between genes coding for pro-inflammatory markers within the experimental groups, a correlation analysis was performed and represented as a heatmap in Figure 8. There were positive associations between the LPS treatment and pro-inflammatory mediator mRNAs in colon, for example, *RANTES*, *IL-12p40*, *IL-18,* and *IL-6* genes (Figure 8). The group fed the SYN diet showed strong similarities to the Control group and had a different distribution of correlations reported to the LPS group (Figure 8).

#### 3.3.2. Effects on Inflammation-Related Protein Expression in Piglets’ Colon

**Figure 9:**
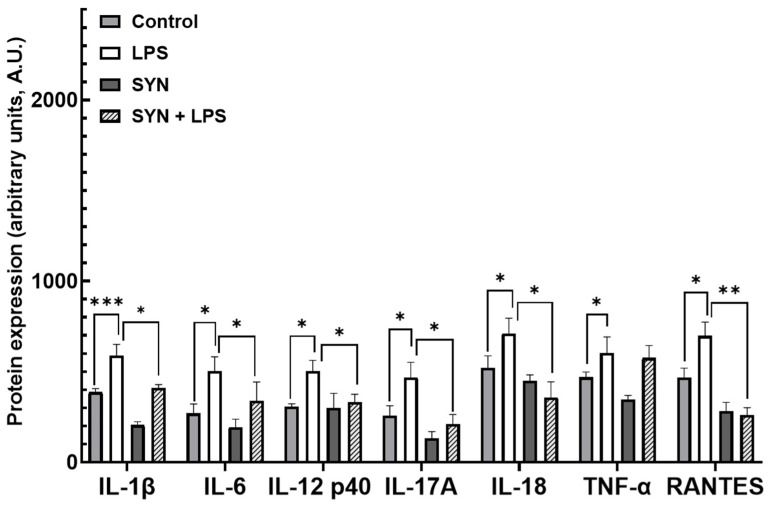
**The effects of the LPS and SYN diet on the selected inflammatory proteins in the colon.** Post-weaned piglets were fed for 21 days with a Control diet (Control and LPS groups) or SYN diet (SYN and SYN+LPS groups). At the end of the feeding trial, piglets from the LPS and SYN+LPS groups were challenged with 80 µg/b.w. LPS. After 3 h, colon samples from all animals were collected and analyzed for IL-1β, IL-6, IL-12 p40, IL-17A, IL-18, TNF-α, and RANTES protein expression by protein arrays as described in the Materials and Methods section. The results are presented as mean ± standard errors. A two-way ANOVA followed by Fisher’s test was used to analyze the effect of experimental treatments on gene expression. Values of *p* < 0.050 were considered significant. *, **, *** Groups with unlike symbols were significantly different (*p* < 0.050 for *; *p* < 0.010 for **, *p* < 0.001 for ***).

In the colon, treatment with LPS induced a strong inflammatory response, 75% (27/36) of inflammation-related proteins being affected (Supplementary Table S6). Among them, IL-1β, IL-6, and RANTES proteins were up-regulated in the colon of LPS-challenged piglets (IL-1β: 1.5-fold increase, *p* < 0.001 vs. Control; IL-6: 1.9-fold increase, *p* = 0.023 vs. Control and RANTES: 1.5-fold increase, *p* = 0.001 vs. Control, Figure 9, Supplementary Table S6). Similarly with their gene expression, 47% (17/36) of inflammatory proteins were unmodified in piglets receiving the SYN diet when compared to the Control (Supplementary Table S6). The protective effects of the SYN diet against LPS-induced inflammatory markers’ expressions were observed in the case of 78% (21/27) of the protein affected by the LPS treatment (IL-1β: -1.4-fold reduction, *p* = 0.050 vs. LPS; IL-6: -1.8-fold reduction, *p* = 0.048 vs. LPS; IL-12 p40: -1.6-fold reduction, *p* = 0.037 vs. LPS; IL-17A: -2.2-fold reduction, *p* = 0.046 vs. LPS; IL-18: -1.9-fold reduction, *p* = 0.004 vs. LPS and RANTES: -2.6-fold reduction, *p* = 0.004 vs. LPS, Figure 9 and Supplementary Table S6). Of these, 52% (14/27) were maintained at the level of the Control (Supplementary Table S6). In piglets’ colon, no significant effects of the experimental diets and of the LPS treatment on anti-inflammatory protein expressions were detected (Supplementary Table S6).

**Figure 10:**
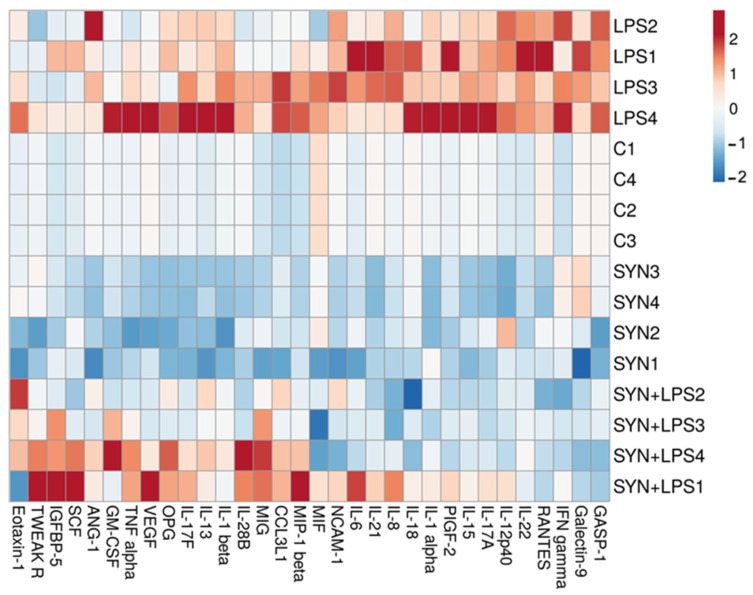
**The effects of the diets and LPS challenge on inflammatory-related proteins in the colon.** The heatmap represents the relative expression levels of genes at the end of the feeding experiment and after 3h of the LPS (LPS and SYN+LPS groups) or saline challenge (SYN group) compared to the unchallenged group (Control group). The magnitude of the protein expression level is represented by a color scale (top), going from low (blue) to high (red).

The heatmap showed positive correlations between pro-inflammatory markers and the LPS challenge, including RANTES, IL-18, and IL-6 (Figure 10), similar to their coding genes. It is noted that the SYN and SYN+LPS groups show similar characteristics, and the Control group has a distinct pattern (Figure 10).

### 3.4. Validation of Gene and Protein Expressions of Pro-Inflammatory Cytokines by ELISA

**Figure 11:**
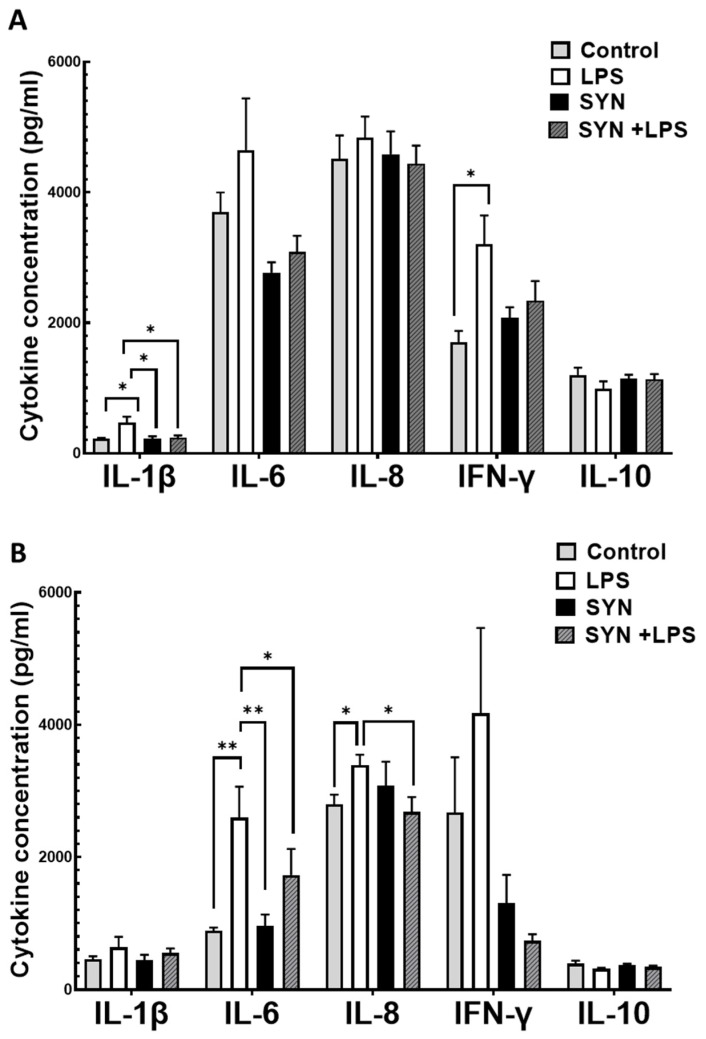
**The effect of SYN and LPS on cytokine concentration in the jejunum (A) and colon (B).** Post-weaned piglets were fed for 21 days with a Control diet (Control and LPS groups) or SYN diet (SYN and SYN+LPS groups). At the end of the feeding trial, piglets from the LPS and SYN+LPS groups were challenged with 80 µg/b.w. LPS. After 3h, the jejunum and colon samples from all animals (n = 8) were collected and analyzed for cytokine concentration using specific kits. Results are presented as means ± standard errors. *, ** Groups with unlike symbols were significantly different (*p* < 0.05 for *; *p* < 0.005 for **).

To validate the results of both qPCR array and protein array analyses, the concentration of selected cytokines (IL-1β, IL-6, IL-8, IFN-γ pro-inflammatory cytokines, and anti-inflammatory cytokine IL-10) was measured by ELISA in the jejunum and colon. The modulation of cytokine concentration by the SYN diet and LPS challenge was similar to that produced on gene expression and on protein expression levels, respectively (Figure 11A,B). Briefly, the LPS treatment induced an increase in the concentration of IL-1β, IL-6, IL-8, and IFN-γ pro-inflammatory cytokines in both the jejunum and colon compared to the Control (Figure 11A,B). The SYN diet alone did not affect cytokine concentration in piglet’s intestines compared to the Control diet (Figure 11A,B) but have positive effects on LPS-induced pro-inflammatory concentration, preventing its increase and maintaining it at the Control level (in jejunum: IL-1β: −49% reduction vs. LPS group, *p* = 0.037; IL-6: −33% reduction vs. LPS group, *p* = 0.118; IL-8: −14% reduction vs. LPS group, *p* = 0.378; IFN-γ: −27% reduction vs. LPS group, *p* = 0.095, Figure 11. A; in colon: L-1β: −14% reduction vs. LPS group, *p* = 0.670; IL-6: −17% reduction vs. LPS group, *p* = 0.179; IL-8: −21% reduction vs. LPS group, *p* = 0.042; IFN-γ: −89% reduction vs. LPS group, *p* = 0.068, Figure 11A). The SYN diet did not affect the concentration of pro-inflammatory cytokines when compared to the Control (Figure 11A,B). In both the jejunum and colon, the SYN diet and LPS challenge had no effect on anti-inflammatory cytokine IL-10 concentration (Figure 11A,B).

### 3.5. Principal Component Analysis

**Figure 12:**
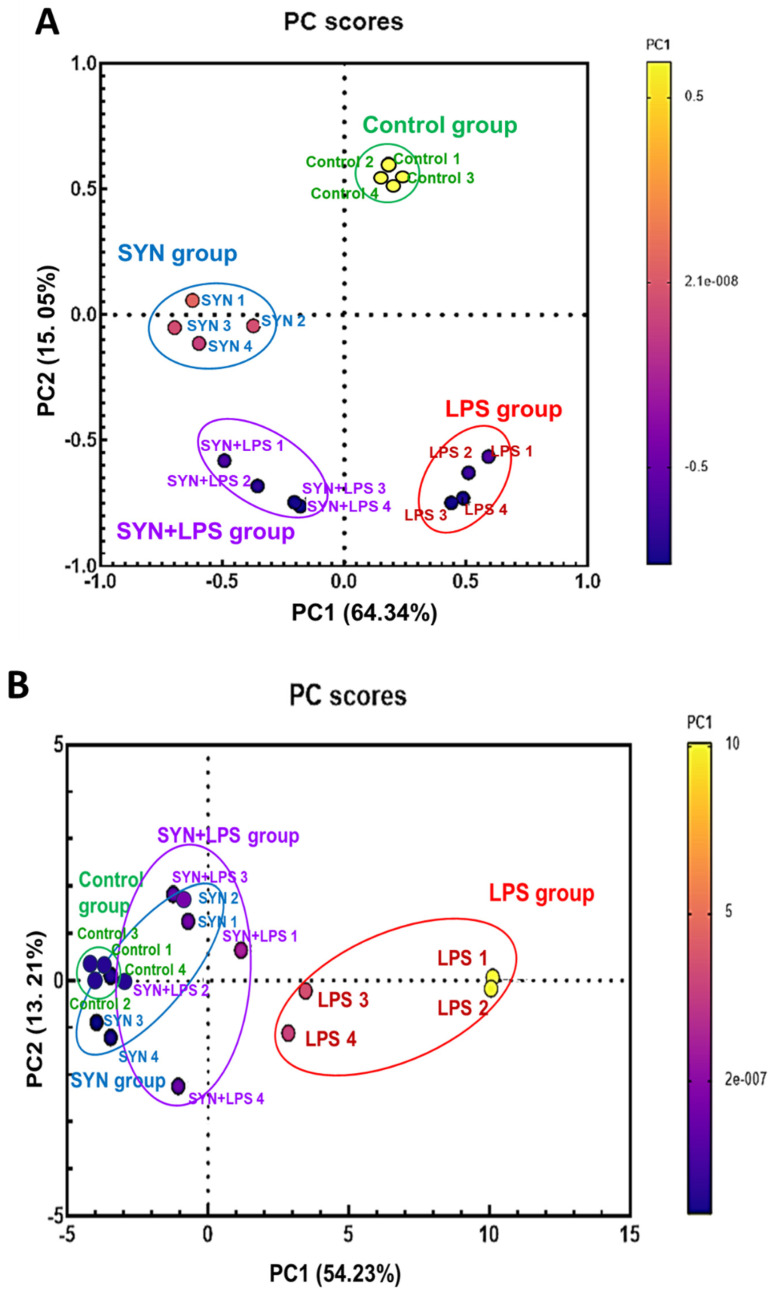
Principal component plots showing the impact of the experimental diets and LPS challenge on the expression of coding for pro-inflammatory markers based on qPCR array data (**A**) and of proteins related to pro-inflammatory response based on protein array data (**B**) in the jejunum. The circles indicate distinct groups (green = Control group, red = LPS group, blue = SYN group, purple = SYN+LPS group) formed by the PCA analysis.

**Figure 13:**
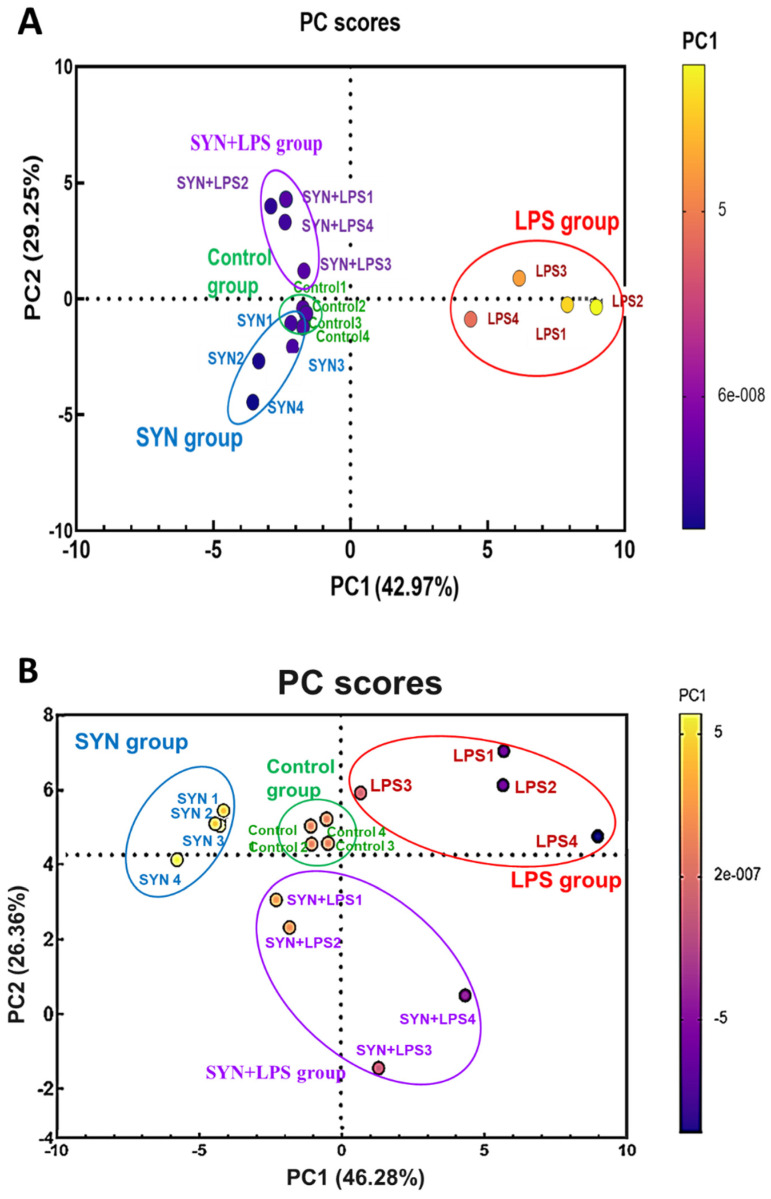
Principal component plots showing the impact of the experimental diets and LPS challenge on the gene expression coding for pro-inflammatory markers based on qPCR array data (**A**) and of proteins related to pro-inflammatory response based on protein array data (**B**) in the colon. The circles indicate distinct groups (green = Control group, red = LPS group, blue = SYN group, purple = SYN+LPS group) formed by the PCA analysis.

Both qPCR and protein array results were subjected to exploratory data analysis (PCA) to obtain a schematic distribution of the gene and protein expression profiles derived from different experimental groups. The PCA analysis showed that LPS-challenged animals formed a distinct cluster (red cluster, Figure 12A and Figure 13A). In the PCA analysis of jejunum genes, PC1 and PC2 explain 64.34% and 15.05% of the total variation, respectively; the protein analysis shows an explanation of 54.23% and 13.21% of total variation in PC1 and PC2, respectively. Biplot representation showed that in both organs, the LPS cluster was associated with the expression of the main pro-inflammatory genes and proteins (red cluster, Figure 12B and Figure 13B). The PCA analysis of pro-inflammatory genes showed that in the jejunum, the SYN and SYN+LPS (blue and purple clusters, Figure 12A) clusters are also separated and did not have similarity with the Control cluster (green cluster, Figure 12A), while the distribution of these groups related to the protein expression showed a great similarity between these three clusters (Control, SYN, and SYN+LPS clusters, Figure 12B). Overall, the Control, SYN, and SYN+LPS clusters are less associated with pro-inflammatory gene expressions in the jejunum (Figure 12B). In the colon, the clusters of unchallenged and synbiotic groups were very similar and showed a low variability between gene and protein expressions (Figure 13A) and a reduced association with pro-inflammatory markers (Figure 13B).

## 4. Discussion

One of the major concerns during weaning and post-weaning piglets is to control the intestinal inflammation by reducing the overexpression of pro-inflammatory markers [36]. As nutritional strategies aimed at preventing/counteracting intestinal disorders induced by weaning stress, the use of feed additives, including synbiotics, is being intensively explored. Therefore, in the present study, we investigated the effects of a synbiotic combination of prebiotics and probiotics as a modulator of intestinal inflammation in piglets after weaning challenged with LPS.

Studies on inflammatory pathologies demonstrated that the lactate dehydrogenase (LDH) enzyme is associated with inflammation and tissue destruction [37]. Plasma levels of LDH and lactate could have a prognostic value for the severity of inflammatory diseases and also for intestinal inflammation [37]. LPS leads to an increase in gluconeogenesis in immune cells by LDH activation; this, in turn, supports the pro-inflammatory response by providing the high energy requirements of inflammation [38]. Based on these data, we expected that LDH would have altered values in the plasma of piglets challenged with LPS. Indeed, our results demonstrated that the LPS treatment was associated with high LDH activity not only in plasma but also in the jejunum and colon of weaned piglets compared to unchallenged animals (+50% increase in plasma, Figure 1, +30% and +45% increase in the jejunum and colon, respectively, Figure 2A,B). Similar results were reported in piglets used as a model for necroptosis, where LPS activates the lactate accumulation and lactase activity, inducing intestinal barrier dysfunction [39]. The induction of LDH activity correlated with LPS-induced damage was also demonstrated in in vivo studies on dextran sulfate sodium (DSS)-treated mice as a model for intestinal inflammation [40] and in vitro studies in the human Caco-2 intestinal cell line [41]. The SYN diet used in our study was able to prevent LPS-induced LDH activity at all levels (plasma and intestinal tissues, Figure 1 and Figure 2A,B), maintaining it at the Control level. Evaluating the impact of synbiotics on LDH leakage in weaned piglets fed with a synbiotic combination of probiotics (*Lactobacillus plantarum* and *Lactobacillus fermentum*) and flaxseed (rich in ω-3 PUFAs), Andrejčáková et al [42] found a reduction in the total LDH leakage and in the LDH isoenzymes concentration in the heart, liver, muscle, and plasma [42]. In mice experimentally infected with *Schistosoma mansoni* and fed with a probiotic (yogurt containing *Lactobacillus casei*, *Lactobacillus plantarum*, *Lactobacillus reuteri*, *Lactobacillus acidophilus*), the LDH activity was reduced in plasma samples [43].

Next, we analyzed the impact of the SYN diet on other markers of intestinal inflammation, using both gene and protein arrays in two segments of intestine; the jejunum, as an organ with an important role in nutrient digestion and absorption; and the colon, with a role in the absorption of fluids and electrolyte and also in providing a physical barrier against microbial invasion [44]. As expected, LPS (80 μg/kg b.w.) was able to induce an inflammatory response in piglets compared to the Control group. It increased the pro-inflammatory cytokines in both the jejunum and colon after 3 h of the *E. coli*-LPS challenge, as demonstrated by both gene and protein analyses. Among the main inflammatory mediators affected by the LPS challenge, the most important were *IL-1β*, *IL-6*, *TNF-α*, *IL-12p40*, *IL-18*, *IFN-γ*, *MIG,* and *RANTES*, overexpressed in both the jejunum and colon. In a similar model of the LPS challenge (100 μg/kg b.w.) in weaning piglets, a maximum peak of *IL-1β* and *IL-6* gene expression was recorded in the jejunum within 2–4 h post-challenge [39]. In addition, we observed that the synbiotic mix had a protective effect on LPS-induced intestinal inflammation, maintaining inflammatory mediators below the LPS group for more than 70% of them, both in the jejunum and colon. Specifically, the SYN diet prevented the LPS-induced increase in master pro-inflammatory cytokines IL-1β, IL-6, TNF-α, and IFN-γ (Figure 3, Figure 4, Figure 5, Figure 6, Figure 7, Figure 8 and Figure 9, Supplementary Tables S3–S6). The SYN diet also maintained at the Control level the expression of IL-12p40, IL-18, and IL-17A cytokines, which are key pro-inflammatory mediators of intestinal inflammation [45]. These results are in accordance with our previous in vitro study on human colon Caco-2 cells challenged with LPS and treated with a synbiotic combination of grape pomace extract and lactobacilli mixture [25], in which the synbiotic reduced, among others, TNF-α, IFN-γ, IL-12p40, and IL-17A mediators. Similar decreases in pro-inflammatory IL-1β, IL-6, and TNF-α in piglets challenged with LPS and enterotoxigenic *Escherichia coli* (ETEC) and fed two synbiotic mixes, including mannan oligosaccharides and *Lactobacillus mucosae* and xylanase and *Bacillus sp.,* respectively, were reported by [20,46]. Data from an in vitro study demonstrated that a synbiotic mix of *Saccharomyces cerevisiae* (var. *boulardii*) and β-galactomannan oligosaccharides decreased the mRNAs of *TNF-α*, *IL-6*, and *GM-CSF* pro-inflammatory cytokines in ETEC-challenged porcine intestinal IPI-2I cells [47].

The inflammatory-inducing effect of LPS was observed not only on cytokines but also on chemokines such as MCP1, MCP2, CXCL12, and RANTES proteins in in vitro models (human monocytes [48] and murine macrophages [49]); these chemokines are overexpressed in chronic intestinal inflammation [50,51]. In our study, the LPS challenge induced an increase in the gene expression of *MCP1*, *MCP2*, *CXCL12,* and *RANTES* chemokines in both the jejunum and colon (Supplementary Tables S3 and S5), whereas the SYN diet prevented the overexpression of these markers in both intestinal segments analyzed. Recent studies demonstrated an immune-stimulating effect of lactobacilli probiotics, the treatment of colonic epithelial cells isolated from mice, with *Lactobacillus rhamnosus GG* inducing the expression of *MCP1* and *RANTES* gene expressions [52], while in our previous in vitro study, a grape pomace extract plus lactobacilli mix combination had similar effects with the SYN diet used in the present study, reducing *MCP-1* and *MCP-2* gene expression in LPS-treated Caco-2 cells [25]. In this cellular model, the mRNA for *RANTES* remained unaffected [25], while in LPS-challenged piglets, the SYN diet reduced the *RANTES* mRNA (Supplementary Tables S3 and S5). The efficient modulation of LPS-induced chemokine gene overexpression in the present study might be attributed to the complex synergism between the prebiotic meals and probiotic lactobacilli mixture.

The activation of inflammatory processes by enteric pathogens such as *Escherichia coli* is driven in intestinal cells by the overexpression of the inducible enzyme COX-2 [53]. Indeed, in the present study, the challenge with LSP induced an increase in *COX2* gene expression in both intestinal segments analyzed (Supplementary Tables S3 and S5). The SYN diet prevented the overexpression of the *COX2* gene, maintaining it at the Control value in the jejunum. Similar results were reported in a cellular model of *E. coli*-challenged Caco-2 cells, where a synbiotic combination of quercetin polyphenol and *Lactobacillus acidophilus LA5* probiotic reduced the *E coli*-induced COX2 protein expression [54].

Regarding the possible mechanism of action induced by probiotics, our previous transcriptomic analysis on the effects of a probiotic lactobacilli mixture (*L. rhamnosus*, *L. plantarum,* and *L. paracasei*) demonstrated that the exposure of intestinal cells to this mixture affected Wnt/β-catenin signaling [55]. This canonical pathway is involved in the regulation of intestinal cell proliferation, differentiation, and homeostasis [56]. In the same way, another study showed that *Bacillus subtilis* can alleviate LPS-induced inflammation and intestinal barrier damage by activating the Wnt/β-catenin pathway [57].

Investigations so far have shown two main mechanisms of action of prebiotics: (i) the regulation of the main signaling pathway involved in the control of inflammation, the Mitogen-activated Kinase (MAPK)/Nuclear factor -kB (NF-kB) pathway [58], and (ii) the modulation of gut microbiota. For example, in an in vitro study on human Caco-2 cells, we demonstrated that a combination of grape pomace polyphenolic extract and lactobacilli mix (*L. rhamnosus*, *L. plantarum,* and *L. paracasei*) attenuated LPS-induced inflammation by downregulating the MAPK/NF-kB pathway [25]. In the same study, we demonstrated that this synbiotic combination also affected the Akt/P70S6K/mTOR signaling pathway [25], involved in modulating immune and inflammatory responses [59]. Our group has demonstrated the effect of prebiotics on this pathway as well as in in vivo studies. Feeding piglets after weaning with a diet containing grape seed meal rich in bioactive compounds suppressed the MAPK/NF-kB pathway induced by dextran sulphate used to simulate intestinal inflammation [60]. Prebiotics can also act as selective modulators of bacterial composition in the gut, positively regulating the non-pathogenic bacteria. For example, a diet including grape pomace rich in polyphenols and fibers promoted the growth of beneficial bacteria in cecum content, improving the piglet’s resistance to disease [61]. Grosu et al. also demonstrated that grape seed meal stimulated the growth of beneficial genera such as *Prevotella* and *Megasphaera* in the gut of dextran sodium sulphate-challenged piglets [62].

To our knowledge, this is the first study to report a broad range of markers modulated by both the LPS challenge and SYN diet in weaning piglets; other similar studies have focused on a few cytokines involved in intestinal inflammation. The pattern of results on a wide spectrum of inflammatory markers (cytokines and chemokines) shown herein indicates that piglets in the LPS group had an increased expression of markers related to intestinal inflammation in both the small intestine (jejunum) and the colon. This could be a consequence of the induction of bacterial infection-associated stress mimicked by the LPS challenge and is not a feature of piglets receiving the SYN diet. This, in turn, may indicate that supplementation with SYN of the piglets’ diet can alleviate intestinal inflammation in *E. coli*-LPS-challenged piglets.

## 5. Conclusions

In conclusion, our results demonstrated that the LPS challenge induced an exacerbated inflammatory response in the jejunum and colon of piglets by increasing inflammation-related mediators, including LDH activity, cytokines (e.g., IL-1β, IL-6, TNF-α), and chemokines (e.g., MIG, RANTES). All these effects were prevented by the SYN diet, which counteracted the overexpression of these pro-inflammatory markers and controlled the amplitude of intestinal inflammation induced by the LPS challenge in piglets. Taken together, these results suggested that weaned piglets fed the synbiotic diet are less susceptible to the negative effects of the LPS challenge, and this could be used as a strategy to alleviate enteric problems and intestinal inflammation in piglets after weaning.

## Figures and Tables

**Table 1:** (A) The composition of bioactive compounds (polyphenols) in the prebiotic mix. (B) The composition of bioactive compounds (fatty acids) in the prebiotic mix. (C) The composition of other bioactive compounds (carbohydrates, organic acids, and microminerals) in the prebiotic mix.

**(A)**	
**Polyphenols (mg/100 g)**	**Prebiotic Mix**
Total polyphenols	3267.9
Catechins	104.70
Caffeic acid	21.75
Gallic acid	12.50
Vanillic acid	3.69
Epicatechin	5.09
p-Coumaric Acid	0.36
Ferulic Acid	0.17
Sinapic Acid	1.29
Rutin	2.96
**(B)**	
**Fatty Acids (g/100 g)**	**Prebiotic Mix**
Total PUFAS	64.37
cis Oleic Acid (C 18:1n9)	15.64
cis Linoleic Acid (C 18:2)	47.71
α-Linolenic Acid (C 18:3n3)	14.84
Eicosanoic Acid (C:20(1n9))	5.18
Octadecatetraenoic Acid (C 18:4n3)	0.55
Eicosadienoic Acid (C 20:2n6)	0.66
Eicosatrienoic Acid (C 20:3n6)	0.33
Erucic Acid (C22 (1n9))	1.02
Eicosapentaenoic (C 20:5n3)	0.29
ω-6 PUFAs	54.47
ω-3 PUFAs	15.68
**(C)**	
**Bioactive Compounds**	**Prebiotic Mix**
**Carbohydrates (mg/100 g)**	
Sucrose	1063.88
Glucose	446.15
Fructose	691.52
**Organic acids (mg/100 g)**	
Succinic acid	598.51
Tartric acid	231.80
Oxalic acid	216.63
**Microminerals (ppm)**	
Copper	5.76
Iron	117.38
Manganese	17.42
Zinc	28.72
Sodium	43.51
**Fiber (%)**	33.61

**Table 2:** The composition of the experimental diets (%).

Ingredients	Control Diet	SYN Diet
Corn	57.43	52.67
Barley	5	5
Wheat	10	10
Soybean meal (45.5% CP)	20	18.7
Gluten	2	2
Powdered milk	2	2
Sunflower oil	0.2	1.2
Monocalcium phosphate	0.1	0.12
Calcium carbonate	1.16	1.12
Salt	0.35	0.35
DL-Methionine, 99% Met	0.14	0.12
L-Lysine -HCl, 78% Lys	0.42	0.42
Choline premix	0.2	0.2
Vitamin mineral premix ^1^	1	1
Prebiotic mix ^2^		5
Probiotic mix ^3^		0.1
**Calculated nutritive value (%)**		
Metabolizable energy (MJ/kg) ^4^	13.60	13.57
Crude protein	18.15	18.12
Crude fat	2.87	2.98
Crude fiber	3.38	4.72
Lysine, total	1.20	1.21
Lysine, digestible	1.02	1.01
Methionine + cystine, total	0.75	0.75
Methionine + cystine, digestible	0.62	0.62
Threonine	0.89	0.87
Tryptophan	0.20	0.19
Valine	0.94	0.91
Calcium	0.75	0.75
Phosphorus, total	0.65	0.66

^1^ Vitamin–mineral premix/kg diet: 10,000 UI vitamin A; 2000 vitamin D3; 30 UI vitamin E; 2 mg vitamin K; 1.96 mg vitamin B1; 3.84 mg vitamin B2; 14.85 mg pantothenic acid; 19.2 mg nicotinic acid; 2.94 mg vitamin B6; 0.98 mg folic acid; 0.03 mg vitamin B12; 0.06 biotin; 24.5 mg vitamin C; 40.3 mg Mn; 100 mg Fe; 100 mg Cu; 100 mg Zn; 0.38 I; 0.23 mg Se; 0.2 g Axtra PHY 5000 L (1000 FTU). ^2^ mix of grape seed meal and camelina meal (3:1 ratio). ^3^ mix of Lactobacilli (Lactobacillus plantarum 0.5 × 10^7^ CFU/g, Lactobacillus paracasei 0.5 × 10^7^ CFU/g, Lactobacillus acidophilus 0.5 × 10^7^ CFU/g). ^4^ Metabolizable energy calculated from the specified raw nutrient content.

## Data Availability

The datasets used and/or analyzed during the current study are available from the corresponding author on reasonable request.

## Supplementary Materials

The following supporting information can be downloaded at https://www.mdpi.com/article/10.3390/ani15131832/s1, Table S1: List and characteristics of target genes detected in qPCR arrays; Table S2: The list of proteins spotted on the membranes of cytokine arrays. Table S3. The effects of the LPS and SYN diet on the genes coding for inflammatory markers in jejunum samples. Table S4. The effects of the SYN diet on the expression of the inflammatory-related proteins in the jejunum of piglets challenged with LPS. Table S5. The effects of the LPS and SYN diet on the genes coding for inflammatory markers in the colon. Table S6. The effects of the SYN diet on the expression of the inflammatory-related proteins in the colon of piglets challenged with LPS.
