# Supplementary material for: Dietary Synbiotic Attenuated the Intestinal Inflammation in Weaned Piglets Challenged with Escherichia coli Lipopolysaccharide
Source: Animals (Basel). 2025 Jun 20;15(13):1832. doi: 10.3390/ani15131832 (PMC12248709; doi:10.3390/ani15131832)
Supplement: Supplementary file 1 [file animals-15-01832-s001.zip › animals-3695295-supplementary.pdf]

Supplementary Table S1. List and characteristics of target genes detected in qPCR array.

No crt	Species	Accession No	Gene abbreviation	Gene name	qPCR amplicon size (bp)
1	Sus scrofa	NM_001302388.2	*IL1B*	Interleukin - 1 beta	162
2	Sus scrofa	NM_214022.1	*TNF*	Tumour necrosis factor – alpha	157
3	Sus scrofa	NM_001005729.1	*IL12A*	Interleukin-12 subunit alpha (IL-12 p35)	113
4	Sus scrofa	NM_001005729.1	*IL17A*	Interleukin-17A	113
5	Sus scrofa	NM_214321.1	*PTGS2*	Prostaglandin-endoperoxide synthase 2	112
6	Sus scrofa	XM_021076655.1	*PTGER4*	Prostaglandin E Receptor 4	103
7	Sus scrofa	NM_214015.1	*TGFB1*	Transforming growth factor beta 1	141
8	Sus scrofa	NM_214192.2	*MMP2*	Matrix metalloproteinase 2	96
9	Sus scrofa	XM_013995919.2	*IL1R1*	Interleukin 1 receptor, type I	162
10	Sus scrofa	NM_213969.1	*TNFRSF1A*	TNF receptor superfamily member 1A	113
11	Sus scrofa	NM_214013.1	*IL12B*	Interleukin-12 subunit beta	106
12	Sus scrofa	XM_013978026.2	*IL17F*	Interleukin-17F	124
13	Sus scrofa	NM_213787.1	*SERPINA3*	Serine (Or Cysteine) Proteinase Inhibitor, Clade A (Alpha-1 Antiproteinase, Antitrypsin), Member 3	153
14	Sus scrofa	NM_001166491.1	*CCL1*	C-C Motif Chemokine Ligand 1	168
15	Sus scrofa	XM_021064293.1	*TGFB2*	Transforming growth factor beta 2	103
16	Sus scrofa	NM_001038004.1	*MMP9*	Matrix metalloproteinase 9	91
17	Sus scrofa	NM_214399.1	*IL6*	Interleukin-6	146
18	Sus scrofa	NM_001097441.2	*TNFRSF1B*	TNF receptor superfamily member 1B	138
19	Sus scrofa	NM_001130236.1	*IL23A*	Interleukin-23 subunit alpha	165
20	Sus scrofa	NM_213997.1	*IL18*	Interleukin-18	134
21	Sus scrofa	NM_001008691.1	*CXCL10*	CXC motif chemokine ligand 10	92
22	Sus scrofa	XM_013997125.2	*RORC*	RAR related orphan receptor C	162
23	Sus scrofa	NM_001009580.1	*CXCL12*	CXC motif chemokine ligand 12	157
24	Sus scrofa	NM_213857.1	*TIMP1*	Metalloproteinase Inhibitor 1	146
25	Sus scrofa	NM_214403.1	*IL6R*	Interleukin 6 receptor	117
26	Sus scrofa	XM_021081528.1	*TNFAIP2*	TNF Alpha Induced Protein 2	96
27	Sus scrofa	XM_021102700.1	*IL20*	Interleukin-20	91
28	Sus scrofa	NM_001137621.1	*IL23R*	Interleukin-23 receptor	144
29	Sus scrofa	NM_214214.1	*CCL2*	C-C motif chemokine ligand 2 (MCP1)	99
30	Sus scrofa	NM_001129946.2	*CCL5*	Chemokine (C-C motif) ligand 5 (RANTES)	160
31	Sus scrofa	NM_213773.1	*CXCR4*	C-X-C chemokine receptor type 4	125
32	Sus scrofa	NM_001145985.1	*TIMP2*	Metalloproteinase Inhibitor 2	123
33	Sus scrofa	NM_214205.1	*IL5*	Interleukin-5	133
34	Sus scrofa	NM_001243634.1	*IL13RA2*	Interleukin-13 receptor subunit alpha-2	168
35	Sus scrofa	NM_214159.1	*PIGR*	Polymeric immunoglobulin receptor	124
36	Sus scrofa	NM_213816.1	*ICAM1*	Intercellular Adhesion Molecule 1	145
37	Sus scrofa	NM_001164515.1	*CCL8*	Chemokine (C-C motif) ligand 8 (MCP2)	91
38	Sus scrofa	NM_001114289.2	*CXCL9*	CXC Motif Chemokine Ligand 9 (MIG)	116
39	Sus scrofa	NM_001246240.1	*IDO1*	Indoleamine 2,3-dioxygenase 1	122
40	Sus scrofa	NM_001007520.1	*IL27*	Interleukin-27	160

Supplementary Table S2: List of proteins spotted on the membranes of cytokine arrays.

No crt	Species	Accession No	Protein abbreviation	Protein name
1	Sus scrofa	F1S1F8	ANG-1	Angiopoietin-1
2	Sus scrofa	A1YM34	CCL3L1	Chemokine (C-C motif) ligand 3-like 1
3	Sus scrofa	Q9XSD9	Decorin	Decorin
4	Sus scrofa	CAB07027.1	Eotaxin-1	Eotaxin-1
5	Sus scrofa	P49157	EPO	Erythropoietin
6	Sus scrofa	C6JWG4	FGF-21	Fibroblast growth factor 21
7	Sus scrofa	O08573.1	Galectin-9	Galectin-9
8	Sus scrofa	Q5JY77.3	GASP-1	G-protein coupled receptor-associated sorting protein 1
9	Sus scrofa	Q29118	GM-CSF	Granulocyte-macrophage colony-stimulating factor
10	Sus scrofa	P49879	IFN-α	Interferon-alpha
11	Sus scrofa	Q29412	IFN-β	Interferon-beta
12	Sus scrofa	P17803	IFN-γ	Interferon-gamma
13	Sus scrofa	P23695	IGF-2	Insulin-like growth factor 2
14	Sus scrofa	Q28985	IGFBP-5	Insulin-Like Growth Factor Binding Protein-5
15	Sus scrofa	Q29055	IL-10	Interleukin-10
16	Sus scrofa	Q29053	IL-12p40	Interleukin-12 subunit beta
17	Sus scrofa	Q95J68	IL-13	Interleukin-13
18	Sus scrofa	Q95253	IL-15	Interleukin-15
19	Sus scrofa	Q60I29	IL-17A	Interleukin-17A
20	Sus scrofa	F1RKV0	IL-17F	Interleukin-17F
21	Sus scrofa	Q9TU73	IL-18	Interleukin-18
22	Sus scrofa	P18430	IL-1 alpha	Interleukin-1 alpha
23	Sus scrofa	P26889	IL-1 beta	Interleukin-1 beta
24	Sus scrofa	Q29056	IL-1 ra	Interleukin-1 receptor antagonist
25	Sus scrofa	Q76LU6	IL-21	Interleukin-21
26	Sus scrofa	K7GM74	IL-22	Interleukin-22
27	Sus scrofa	D0FHA1	IL-28B	Interleukin-28 subunit B
28	Sus scrofa	Q04745	IL-4	Interleukin-4
29	Sus scrofa	P26893	IL-6	Interleukin-6
30	Sus scrofa	P26894	IL-8	Interleukin-8
31	Sus scrofa	P01315	Insulin	Insulin
32	Sus scrofa	P80928	MIF	Macrophage migration inhibitory factor
33	Sus scrofa	B0FYK2	MIG	Monokine induced by interferon-gamma
34	Sus scrofa	Q711P4	MIP-1 beta	Macrophage Inflammatory Protein-1 beta
35	Sus scrofa	F1SM72	NCAM-1	Neural cell adhesion molecule-1
36	Sus scrofa	F1S278	OPG	Osteoprotegerin
37	Sus scrofa	P20034	PDGF-BB	Platelet Derived Growth Factor BB
38	Sus scrofa	K7GPI3	PECAM-1	Platelet endothelial cell adhesion molecule-1
39	Sus scrofa	Q58IV6	PIGF-2	heparin-binding form of placenta growth factor
40	Sus scrofa	Q29288	RANTES	Regulated on activation, normal T cell expressed and secreted
41	Sus scrofa	Q29030	SCF	SKP1, CUL1, F-box protein

42	Sus scrofa	P01135	TGF-alpha	Transforming growth factor alpha
43	Sus scrofa	P07200	TGF beta 1	Transforming growth factor beta 1
44	Sus scrofa	P35624	TIMP-1	Metalloproteinase Inhibitor 1
45	Sus scrofa	C0JPM4	TIMP-2	Metalloproteinase Inhibitor 2
46	Sus scrofa	P23563	TNF alpha	Tumor necrosis factor alpha
47	Sus scrofa	B7SLY2	TWEAK R	Tumor necrosis factor receptor superfamily member 12A
48	Sus scrofa	P49151	VEGF	Vascular endothelial growth factor

Supplementary Table S3. The effects of LPS and SYN diet on the genes coding for inflammatory markers in jejunum samples.

Gene name	Experimental group*			
	Control	LPS	SYN	SYN+LPS
	Fc ** (Mean ± SEM)	Fc ** (Mean ± SEM)	Fc ** (Mean ± SEM)	Fc ** (Mean ± SEM)
**Genes coding for pro-inflammatory mediators**				
**IL-1β**	1.00 ± 0.00 [b]	3.69 ± 0.36 [a]	0.75 ± 0.28 [b]	1.01 ± 0.32 [b]
**IL-5**	1.00 ± 0.00 [b]	5.42 ± 0.35 [a]	0.44 ± 0.12 [c]	0.74 ± 0.43 [b,c]
**IL-6**	1.00 ± 0.00 [b]	10.37 ± 1.46 [a]	0.39 ± 0.09 [c]	1.20 ± 0.08 [b]
**IL-12p35**	1.00 ± 0.00 [b]	3.78 ± 0.65 [a]	0.42 ± 0.15 [c]	0.74 ± 0.10 [b]
**IL-12 p40**	1.00 ± 0.00 [b]	3.75 ± 1.33 [a]	0.64 ± 0.10 [c]	3.38 ± 0.53 [a]
**IL17A**	1.00 ± 0.00 [b]	1.60 ± 0.66 [a]	0.29 ± 0.03 [c]	0.71 ± 0.17 [b]
**IL17F**	1.00 ± 0.00	1.33 ± 0.28	1.64 ± 0.26	1.45 ± 0.13
**IL-18**	1.00 ± 0.00 [c]	16.64 ± 0.80 [a]	0.88 ± 0.27 [c]	2.49 ± 0.77 [b]
**IL-20**	1.00 ± 0.00 [b]	4.59 ± 0.81 [a]	0.50 ± 0.11 [c]	0.84 ± 0.17 [b]
**IL-23**	1.00 ± 0.00 [b]	1.69 ± 0.29 [a]	1.33 ± 0.23 [b]	1.23 ± 0.40 [b]
**IL-27**	1.00 ± 0.00 [b]	1.67 ± 0.32 [a]	0.07 ± 0.01 [d]	0.48 ± 0.05 [c]
**TNF-α**	1.00 ± 0.00 [b]	4.28 ± 0.33 [a]	1.08 ± 0.21 [b]	1.09 ± 0.24 [b]
**IL1R1**	1.00 ± 0.00 [b]	5.94 ± 0.79 [a]	0.28 ± 0.02 [c]	0.62 ± 0.12 [b]
**IL-6R**	1.00 ± 0.00 [a]	1.29 ± 0.06 [a]	1.23 ± 0.69 [a]	1.29 ± 0.56 [a]
**IL13RA2**	1.00 ± 0.00 [b]	2.59 ± 0.32 [a]	1.07 ± 0.39 [b]	0.58 ± 0.08 [c]
**IL23R**	1.00 ± 0.00 [a]	0.62 ± 0.09 [b]	0.80 ± 0.28 [a]	0.73 ± 0.08 [b]
**s TNF RI**	1.00 ± 0.00 [b]	1.75 ± 0.48 [a]	0.13 ± 0.04 [c]	0.63 ± 0.13 [b]
**s TNF RII**	1.00 ± 0.00 [b]	2.73 ± 0.66 [a]	0.88 ± 0.19 [b]	0.94 ± 0.20 [b]
**TNFAIP2**	1.00 ± 0.00 [b]	1.99 ± 0.83 [a]	1.68 ± 0.70 [a]	0.87 ± 0.22 [b]
**MMP2**	1.00 ± 0.00 [b]	3.07 ± 0.32 [a]	1.25 ± 0.28 [b]	1.09 ± 0.18 [b]
**MMP9**	1.00 ± 0.00 [b]	2.34 ± 0.36 [a]	1.10 ± 0.12 [b]	1.88 ± 0.22 [a]
**MCP-1**	1.00 ± 0.00 [b]	1.75 ± 0.15 [a]	1.00 ± 0.27 [b]	0.65 ± 0.06 [c]
**MCP-2**	1.00 ± 0.00 [b]	3.51 ± 0.78 [a]	0.39 ± 0.18 [c]	0.26 ± 0.10 [c]
**COX2**	1.00 ± 0.00 [b]	6.79 ± 1.01 [a]	0.96 ± 0.33 [b]	0.96 ± 0.26 [b]
**PTGER4**	1.00 ± 0.00 [b]	1.28 ± 0.46 [a]	0.31 ± 0.06 [c]	0.46 ± 0.11 [c]
**SERPINA3**	1.00 ± 0.00	0.76 ± 0.15	1.37 ± 0.32	0.91 ± 0.10
**I-309**	1.00 ± 0.00 [b]	3.82 ± 0.79 [a]	0.18 ± 0.08 [c]	1.05 ± 0.07 [b]
**IP-10**	1.00 ± 0.00 [c]	8.13 ± 1.49 [a]	1.51 ± 0.10 [b]	5.68 ± 0.99 [a]
**RORC**	1.00 ± 0.00 [b]	2.50 ± 0.91 [a]	1.98 ± 0.51 [b]	2.10 ± 0.24 [a]
**CXCL12**	1.00 ± 0.00 [b]	2.57 ± 0.34 [a]	0.45 ± 0.10 [c]	1.89 ± 0.35 [a,b]
**RANTES**	1.00 ± 0.00 [b]	6.26 ± 0.49 [a]	0.83 ± 0.07 [b]	1.36 ± 0.24 [b]
**CXCR4**	1.00 ± 0.00 [c]	5.81 ± 0.76 [a]	0.23 ± 0.13 [d]	2.13 ± 0.32 [b]
**PIGR**	1.00 ± 0.00 [a]	0.34 ± 0.08 [b]	0.87 ± 0.22 [a]	0.61 ± 0.42 [a]
**ICAM1**	1.00 ± 0.00 [c]	8.65 ± 1.07 [a]	1.88 ± 0.26 [b]	3.29 ± 0.61 [b]
**MIG**	1.00 ± 0.00 [b]	9.10 ± 1.25 [a]	1.11 ± 0.27 [b]	0.78 ± 0.14 [b]
**IDO1**	1.00 ± 0.00 [b]	3.23 ± 0.82 [a]	0.61 ± 0.19 [b]	1.62 ± 0.36 [b]
**Genes coding for anti-inflammatory mediators**				
**TGF-β1**	1.00 ± 0.00	0.79 ± 0.09	0.66 ± 0.06	0.64 ± 0.05
**TGF-β2**	1.00 ± 0.00	1.25 ± 0.25	1.71 ± 0.39	1.58 ± 0.65
**TIMP1**	1.00 ± 0.00	0.89 ± 0.01	1.15 ± 0.28	1.02 ± 0.22
**TIMP2**	1.00 ± 0.00	0.96 ± 0.24	1.15 ± 0.28	1.33 ± 0.33

*post-weaned piglets were fed for 21 days with a control diet (control and LPS groups) or SYN diet (SYN and SYN+LPS groups). At the end of the feeding trial, piglets from the LPS and SYN+LPS groups were challenged with 80μg/b.w. LPS. After 3h, jejunum samples from all animals were collected and analyzed for gene expression by qPCR array (n=4).
** the results were expressed as fold changes (Fc) compared to the Control group and are presented as average ± standard error of the mean (mean ± SEM). The effects of experimental diets and of LPS challenge on the mRNA expression were analyzed using two-way Anova and Fisher tests. Values of $p < 0.05$ were considered significant.
[a,b,c] Mean values within a row with unlike superscript letters were significantly different (p<0.05).

Supplementary Table S4. The effects of SYN diet on the expression of the inflammatory-related proteins in the jejunum of piglets challenged with LPS.

Protein name	Experimental group*			
	Control	LPS	SYN	SYN+LPS
	A.U. ** (Mean ± SEM)	A.U. ** (Mean ± SEM)	A.U. ** (Mean ± SEM)	A.U. ** (Mean ± SEM)
**Pro-inflammatory mediators**				
**ANG-1**	389.5 ± 65.5 [c]	1371.2 ± 231.1 [a]	719.5 ± 30.8 [b]	821.8 ± 64.1 [b]
**CCL3L1**	1225.8 ± 528.2 [b]	2912.7 ± 191.8 [a]	1533.4 ± 81.1 [b]	1480.4 ± 393.9 [b]
**Eotaxin-1**	707.9 ± 127.7 [c]	2056.4 ± 307.1 [a]	814.7 ± 126.3 [c]	1409.0 ± 78.9 [b]
**Galectin-9**	197.2 ± 34.8 [b]	601.1 ± 137.1 [a]	581.4 ± 178.5 [a,b]	408.3 ± 45.0 [a,b]
**GASP-1**	282.4 ± 113.0 [c]	1874.4.0 ± 490.0 [a]	547.7 ± 166.7 [b]	793.6 ± 42.9 [b]
**GM-CSF**	171.5 ± 58.4 [c]	979.8 ± 232.5 [a]	206.9 ± 36.4 [c]	621.4 ± 109.9 [b]
**IFN-γ**	229.3 ± 85.2 [b]	593.8 ± 57.5 [a]	299.1 ± 82.0 [b]	510.70 ± 107.0 [a]
**IGFBP-5**	198.7 ± 96.0 [b]	521.3.1 ± 73.2 [a]	148.6 ± 34.4 [b]	394.8 ± 178.0 [a,b]
**IL-12p40**	289.7 ± 64.6 [b]	922.8 ± 80.8 [a]	356.8 ± 141.7 [b]	402.8 ± 76.4 [b]
**IL-13**	726.2 ± 190.7 [b]	1644.6 ± 370.3 [a]	997.8 ± 315.7 [b]	522.4 ± 33.5 [b]
**IL-15**	280.5 ± 74.4 [b]	700.9 ± 100.7 [a]	188.8 ± 31.2 [b]	293.6 ± 121.5 [b]
**IL-17A**	220.0 ± 30.7 [b]	533.3 ± 77.2 [a]	270.3 ± 45.2 [b]	281.2 ± 80.9 [b]
**IL-17F**	184.7 ± 86.3 [b]	606.5 ± 48.2 [a]	201.6 ± 54.8 [b]	247.2 ± 78.6 [b]
**IL-18**	280.3 ± 57.5 [c]	1161.5 ± 279.9 [a]	240.4 ± 82.0 [c]	839.2 ± 171.3 [b]
**IL-1α**	196.2 ± 67.3 [c]	798.5 ± 125.6 [a]	412.7 ± 67.3 [b]	413.5 ± 37.7 [b]
**IL-1β**	216.9 ± 77.3 [b]	821.9 ± 100.5 [a]	301.99 ± 115.6 [b]	308.5 ± 102.6 [b]
**IL-21**	156.3 ± 52.7 [b]	435.6 ± 63.8 [a]	221.5 ± 36.2 [b]	342.6 ± 68.4 [a]
**IL-22**	165.9 ± 41.2 [c]	646.8 ± 160.3 [a]	232.9 ± 63.9 [c]	411.4 ± 82.8 [b]
**IL-28B**	1482.3 ± 734.0 [a]	2075.2 ± 314.5 [a]	821.3 ± 160.4 [b]	1392.1 ± 161.7 [a]
**IL-6**	295.5 ± 80.7 [c]	1083.4 ± 380.5 [a]	287.6 ± 42.0 [c]	565.9 ± 110.0 [b]
**IL-8**	1220.1 ± 595.5 [b]	3974.7 ± 658.9 [a]	925.3 ± 121.9 [b]	1356.4 ± 226.1 [b]
**MIF**	2253.5 ± 717.5	2977.2 ± 513.9	2127.6 ± 318.5	2609.7 ± 211.5
**MIG**	4679.9 ± 635.7 [b]	8559.5 ± 122.9 [a]	6299.2 ± 337.8 [a,b]	3565.2 ± 136.5 [b]
**MIP-1β**	1778.7 ± 369.5 [c]	8256.5 ± 307.5 [a]	3903.8 ± 144.9 [b]	2554.9 ± 260.2 [b,c]
**NCAM-1**	308.6 ± 130.4 [c]	2278.9 ± 371.2 [a]	779.9 ± 191.5 [b]	905.4 ± 180.4 [b]
**OPG**	275.3 ± 76.8 [b]	699.0 ± 134.3 [a]	245.6 ± 63.3 [b]	444.4 ± 98.4 [a,b]
**PIGF-2**	171.9 ± 74.4 [b]	644.1 ± 131.3 [a]	219.7 ± 41.9 [b]	210.9 ± 39.3 [b]
**RANTES**	420.1 ± 82.8 [b]	1069.5 ± 228.1 [a]	286.2 ± 58.7 [c]	215.6 ± 76.1 [c]
**SCF**	581.3 ± 54.2 [b]	1621.1 ± 290.2 [a]	435.4 ± 53.7 [b]	479.1 ± 59.8 [b]
**TNF-α**	350.4 ± 73.2 [c]	1390.0.1 ± 185.7 [a]	621.5 ± 104.6 [b]	601.9 ± 58.1 [b]
**TWEAK R**	703.6 ± 30.0	732.6 ± 32.5	480.9 ± 107.4	553.0 ± 110.2
**VEGF**	281.3 ± 53.8 [c]	1356.5 ± 132.0 [a]	465.2 ± 97.1 [b]	437.3 ± 37.9 [b,c]
**Decorin**	25527.3 ± 2719.7 [a]	23939.7 ± 2911.7[a]	24404.7 ± 1436.9[a]	33879.0 ± 3934.3 [a]
**IFN-α**	429.3 ± 100.2 [b]	965.5 ± 195.2 [a]	282.7 ± 41.4 [b]	838.3 ± 72.9 [a]
**IFN-β**	276.2 ± 74.2 [b]	610.9 ± 58.9 [a]	304.8 ± 63.2 [b]	621.9 ± 96.3 [a]
**IGF-2**	442.5 ± 126.5 [c]	1832.0 ± 382.4 [a]	527.8 ± 88.8 [c]	1060.4 ± 134.1 [b]
**Anti-inflammatory mediators**				
**EPO**	392.7 ± 81.2 [b]	597.7 ± 108.5 [a]	260.1 ± 55.0 [b]	475.9 ± 124.7 [b]
**IL-4**	370.8 ± 61.5	468.7.2 ± 90.4	259.1 ± 22.6	263.7 ± 64.2
**IL-10**	289.7 ± 64.6 [b]	753.9 ± 62.4 [a]	441.3 ± 56.5 [b]	557.5 ± 138.2 [a,b]
**IL-1ra**	489.6 ± 16.8	760.3 ± 89.3	360.3 ± 170.4	894.1 ± 93.2
**Insulin**	319.1 ± 44.2 [b]	454.3 ± 16.0 [a]	207.6 ± 55.9 [b]	380.2 ± 115.8 [b]

**PDGF-BB**	269.9 ± 48.5	299.7 ± 61.1	344.1 ± 54.4	351.9 ± 108.4
**PECAM-1**	646.3 ± 3 [a]	849.2 ± 62.4	590.4 ± 132.2	848.7 ± 152.1
**TGF-α**	179.9 ± 49.0 [b]	325.2 ± 36.5 [a]	266.1 ± 76.6 [a,b]	424.5 ± 101.1 [a]
**TGF-β1**	217.3 ± 85.4 [b]	455.0 ± 95.9 [a]	286.6 ± 81.4 [b]	385.4 ± 48.0 [a]
**TIMP-1**	1310.6 ± 38.7	1614.5 ± 185.9	1506.9 ± 121.9	1727.6 ± 101.2
**TIMP-2**	4951.5 ± 1196.2	5112.4 ± 556.9	5966.6 ± 937.6	6602.7 ± 1028.3
**FG21**	387.6 ± 84.5 [a]	492.9 ± 46.5 [a]	174.9 ± 71.4 [b]	494.4 ± 153.1 [a,b]

*post-weaned piglets were fed for 21 days with a control diet (control and LPS groups) or SYN diet (SYN and SYN+LPS groups). At the end of the feeding trial, piglets from the LPS and SYN+LPS groups were challenged with 80µg/b.w. LPS. After 3h, jejunum samples from all animals were collected and analyzed for inflammatory markers expression by protein array (n=4).

** the results were expressed as arbitrary units (A.U.) and are presented as average ± standard error of the mean (mean ± SEM). The effects of experimental diets and of LPS challenge on the protein expression were analyzed using two-way Anova and Fisher tests. Values of $p < 0.05$ were considered significant.

[a,b,c] Mean values within a row with unlike superscript letters were significantly different ($p<0.05$).

Supplementary Table S5. The effects of LPS and SYN diet on the genes coding for inflammatory markers in the colon.

Gene name	Experimental group*			
	Control	LPS	SYN	SYN+LPS
	Fc ** (Mean ± SEM)	Fc ** (Mean ± SEM)	Fc ** (Mean ± SEM)	Fc ** (Mean ± SEM)
	**Genes coding for pro-inflammatory mediators**			
**IL1β**	1.00 ± 0.00 [b]	14.22 ± 1.22 [a]	1.27 ± 0.34 [b]	0.84 ± 0.38 [b]
**IL-5**	1.00 ± 0.00 [a]	0.60 ± 0.12 [a]	1.01 ± 0.25 [a]	0.43 ± 0.10 [b]
**IL-6**	1.00 ± 0.00 [b]	10.40 ± 1.16 [a]	0.51 ± 0.14 [c]	0.65 ± 0.23 [b]
**IL-12p35**	1.00 ± 0.00 [b]	2.92 ± 0.42 [a]	0.26 ± 0.07 [c]	0.97 ± 0.18 [b]
**IL-12 p40**	1.00 ± 0.00 [b]	1.59 ± 0.42 [a]	0.78 ± 0.18 [c]	0.73 ± 0.24 [b]
**IL17A**	1.00 ± 0.00 [b]	4.16 ± 0.89 [a]	0.27 ± 0.07 [c]	0.99 ± 0.16 [b]
**IL17F**	1.00 ± 0.00 [b]	1.92 ± 0.27 [a]	1.09 ± 1.04 [b]	0.71 ± 0.16 [b]
**IL-18**	1.00 ± 0.00 [b]	9.67 ± 1.49 [a]	1.15 ± 0.90 [b]	0.36 ± 0.06 [c]
**IL-20**	1.00 ± 0.00 [b]	0.69 ± 0.08 [a]	0.99 ± 0.20 [c]	0.68 ± 0.14 [b]
**IL-23**	1.00 ± 0.00	0.74 ± 0.11	1.51 ± 0.19	1.04 ± 0.02
**IL-27**	1.00 ± 0.00 [a]	0.66 ± 0.16 [a]	0.06 ± 0.02 [b]	0.05 ± 0.01 [b]
**TNF-α**	1.00 ± 0.00 [b]	5.34 ± 0.70 [a]	1.33 ± 0.13 [b]	1.11 ± 0.13 [b]
**IL1R1**	1.00 ± 0.00 [a]	0.99 ± 0.18 [a]	0.32 ± 0.11 [b]	0.71 ± 0.09 [a]
**IL-6R**	1.00 ± 0.00	1.47 ± 0.25	0.84 ± 0.24	1.26 ± 0.42
**IL13RA2**	1.00 ± 0.00 [a]	0.56 ± 0.06 [b]	1.17 ± 0.22 [a]	0.73 ± 0.20 [a]
**IL23R**	1.00 ± 0.00 [a]	0.48 ± 0.11 [b]	1.28 ± 0.18 [a]	0.35 ± 0.05 [b]
**s TNF RI**	1.00 ± 0.00 [a]	1.40 ± 0.52 [a]	0.23 ± 0.05 [b]	1.09 ± 0.23 [a]
**s TNF RII**	1.00 ± 0.00 [a]	0.92 ± 0.18 [a]	0.81 ± 0.06 [a]	0.55 ± 0.06 [b]
**TNFAIP2**	1.00 ± 0.00 [b]	0.98 ± 0.23 [b]	3.86 ± 1.77 [a]	0.59 ± 0.14 [c]
**MMP2**	1.00 ± 0.00 [b]	2.24 ± 0.28 [a]	1.33 ± 0.26 [b]	1.01 ± 0.29 [b]
**MMP9**	1.00 ± 0.00 [b]	1.59 ± 0.31 [a]	1.58 ± 0.31 [a]	1.48 ± 0.31 [a]
**MCP-1**	1.00 ± 0.00 [b]	5.82 ± 1.40 [a]	1.22 ± 0.18 [b]	0.76 ± 0.06 [c]
**MCP-2**	1.00 ± 0.00 [b]	5.63 ± 1.50 [a]	0.88 ± 0.48 [b]	0.17 ± 0.10 [c]
**COX2**	1.00 ± 0.00 [b]	11.04 ± 1.33 [a]	1.35 ± 0.24 [b]	1.93 ± 0.42 [b]
**PTGER4**	1.00 ± 0.00 [a]	0.84 ± 0.24 [a]	0.90 ± 0.17 [a]	0.56 ± 0.09 [b]
**SERPINA3**	1.00 ± 0.00 [b]	0.69 ± 0.16 [b]	2.54 ± 0.41 [a]	3.57 ± 1.04 [a]
**I-309**	1.00 ± 0.00 [c]	8.81 ± 1.84 [a]	1.59 ± 0.31 [c]	5.30 ± 1.07 [b]
**IP-10**	1.00 ± 0.00 [c]	2.24 ± 0.44 [b]	4.78 ± 0.62 [a]	1.36 ± 0.25 [c]
**RORC**	1.00 ± 0.00 [a]	0.74 ± 0.08 [a]	1.03 ± 0.11 [b]	1.49 ± 0.34 [a]
**CXCL12**	1.00 ± 0.00 [b]	9.83 ± 2.45 [a]	0.63 ± 0.12 [c]	0.40 ± 0.04 [c]
**RANTES**	1.00 ± 0.00 [b]	6.91 ± 1.20 [a]	1.34 ± 0.35 [b]	1.55 ± 0.26 [b]
**CXCR4**	1.00 ± 0.00 [a]	0.73 ± 0.35 [a]	1.17 ± 0.19 [a]	0.57 ± 0.10 [b]
**PIGR**	1.00 ± 0.00 [b]	0.67 ± 0.18 [b]	1.79 ± 0.48 [a]	0.47 ± 0.04 [c]
**ICAM1**	1.00 ± 0.00 [c]	4.72 ± 0.55 [a]	2.93 ± 0.51 [b]	2.32 ± 0.26 [b]
**MIG**	1.00 ± 0.00 [b]	1.66 ± 0.32 [a]	1.11 ± 0.15 [b]	2.39 ± 0.02 [a]

**IDO1**	1.00 ± 0.00 [b]	1.43 ± 0.14 [a]	1.56 ± 0.49 [b]	0.81 ± 0.25 [b]
		**Genes coding for anti-inflammatory mediators**		
**TGF-β1**	1.00 ± 0.00 [a]	0.95 ± 0.15 [a]	0.69 ± 0.12 [b]	0.56 ± 0.08 [c]
**TGF-β2**	1.00 ± 0.00 [b]	0.91 ± 0.16 [b]	3.80 ± 0.31 [a]	2.07 ± 0.44 [b]
**TIMP1**	1.00 ± 0.00 [a]	0.51 ± 0.07 [b]	3.19 ± 0.96 [a]	0.61 ± 0.08 [a]
**TIMP2**	1.00 ± 0.00 [a]	0.30 ± 0.00 [b]	3.18 ± 0.72 [a]	2.27 ± 0.92 [a]

*post-weaned piglets were fed for 21 days with a control diet (control and LPS groups) or SYN diet (SYN and SYN+LPS groups). At the end of the feeding trial, piglets from the LPS and SYN+LPS groups were challenged with 80μg/b.w. LPS. After 3h, colon samples from all animals were collected and analyzed for gene expression by qPCR array (n=4).

** the results were expressed as fold changes (Fc) compared to the Control group and are presented as average ± standard error of the mean (mean ± SEM). The effects of experimental diets and of LPS challenge on the mRNA expression were analyzed using two-way Anova and Fisher tests. Values of $p < 0.05$ were considered significant.

[a,b,c] Mean values within a row with unlike superscript letters were significantly different (p<0.05).

Supplementary Table S6. The effects of SYN diet on the expression of the inflammatory-related proteins in the colon of piglets challenged with LPS.

Protein name	Experimental group*			
	**Control**	**LPS**	**SYN**	**SYN+LPS**
	**A.U. ** (Mean ± SEM)**	**A.U. ** (Mean ± SEM)**	**A.U. ** (Mean ± SEM)**	**A.U. ** (Mean ± SEM)**
		**Pro-inflammatory mediators**		
**ANG-1**	828.1 ± 24.5 [a]	975.3 ± 67.1 [a]	453.7 ± 59.0 [b]	896.3 ± 95.1 [a]
**CCL3L1**	863.1 ± 130.6 [b]	2142.0 ± 412.1 [a]	914.1 ± 169.9 [b]	1933.2 ± 202.6 [a]
**Eotaxin-1**	641.7 ± 98.6	790.0 ± 75.6	549.7 ± 76.8	791.3 ± 141.5
**Galectin-9**	195.0 ± 22.2 [b]	247.7 ± 22.7 [a]	169.1 ± 51.5 [b]	124.6 ± 8.5 [c]
**GASP-1**	453.6 ± 46.7 [b]	690.8 ± 51.0 [a]	262.3 ± 77.7 [b]	291.2 ± 52.9 [b]
**GM-CSF**	352.5 ± 38.2 [a]	497.3 ± 129.3 [a]	181.0 ± 28.1 [b]	535.2 ± 165.2 [a]
**IFN-γ**	105.5 ± 12.0 [b]	296.2 ± 37.2 [a]	188.9 ± 16.9 [b]	109.4 ± 33.3 [b]
**IGFBP-5**	137.2 ± 12.5 [b]	206.1 ± 0.79 [a]	137.9 ± 15.3 [b]	317.4 ± 60.2 [a]
**IL-12p40**	306.3 ± 17.1 [b]	504.1 ± 59.4 [a]	301.0 ± 80.0 [b]	331.0 ± 44.6 [b]
**IL-13**	525.8 ± 64.6 [c]	1092.3 ± 140.9 [a]	270.0 ± 57.4 [d]	847.8 ± 73.1 [b]
**IL-15**	345.8 ± 59.5 [b]	638.6 ± 87.3 [a]	142.0 ± 27.3 [c]	268.0 ± 46.9 [b]
**IL-17A**	256.6 ± 55.7 [b]	469.0 ± 83.1 [a]	132.2 ± 37.5 [c]	210.2 ± 54.1 [b,c]
**IL-17F**	268.9 ± 52.8 [a]	464.7 ± 86.1 [a]	100.9 ± 8.3 [b]	327.4 ± 67.9 [a]
**IL-18**	521.4 ± 66.7 [b]	710.3 ± 84.9 [a]	450.4 ± 32.2 [b]	358.1 ± 86.5 [b]
**IL-1α**	416.6 ± 81.6 [a]	668.9 ± 134.0 [a]	267.8 ± 62.3 [b]	392.6 ± 53.2 [a,b]
**IL-1β**	387.6 ± 19.3 [b]	589.1 ± 62.2 [a]	205.9 ± 18.6 [c]	410.0 ± 19.9 [b]
**IL-21**	290.8 ± 68.6 [b]	550.9 ± 103.5 [a]	50.3 ± 17.6 [c]	225.1 ± 82.2 [b]
**IL-22**	169.8 ± 40.8 [c]	656.2 ± 126.3 [a]	107.7 ± 11.4 [c]	260.0 ± 26.8 [b]
**IL-28B**	904.9 ± 205.6	1029.9 ± 118.2	682.2 ± 38.7	1074.1 ± 226.3
**IL-6**	269.5 ± 53.5 [b]	504.0 ± 78.7 [a]	192.8 ± 45.5 [b]	340.5 ± 103.4 [a]
**IL-8**	548.9 ± 37.9 [b]	809.8 ± 80.9 [a]	427.7 ± 13.3 [c]	503.6 ± 141.0 [a,b]
**MIF**	2095.7 ± 150.3	2041.8 ± 209.3	1743.1 ± 151.0	1551.3 ± 208.3
**MIG**	3125.7 ± 411.8 [c]	5775.0 ± 744.7 [b]	2692.5 ± 694.7[c]	8009.1 ± 1035.7[a]
**MIP-1 β**	1527.7 ± 345.1 [b]	5671.1 ± 943.8 [a]	1373.1 ± 146.4[b]	5151.5 ± 1411.6 [a]
**NCAM-1**	788.9 ± 92.4 [b]	1157.3 ± 66.5 [a]	464.4 ± 55.1 [c]	753.3 ± 148.7 [b]
**OPG**	183.3 ± 38.3 [a]	270.6 ± 69.5 [a]	64.1 ± 7.2 [b]	303.0 ± 59.4 [a]
**PIGF-2**	727.4 ± 55.1 [b]	1632.9 ± 238.3 [a]	424.1 ± 56.7 [c]	664.1 ± 212.6 [b]
**RANTES**	468.3 ± 51.7 [b]	698.1 ± 75.5 [a]	282.2 ± 49.2 [c]	261.9 ± 40.0 [c]
**SCF**	450.3 ± 52.2 [c]	656.8 ± 106.3 [b]	389.1 ± 64.5 [c]	839.6 ± 306.4 [a]
**TNF-α**	469.4 ± 30.0 [a]	694.1 ± 88.5 [a]	345.5 ± 24.2 [b]	576.7 ± 68.3 [a]
**TWEAK R**	357.4 ± 27.6	319.4 ± 58.5	266.7 ± 67.0	571.5± 119.8
**VEGF**	504.9 ± 53.1[a]	654.6 ± 141.5[a]	189.9 ± 46.1 [b]	577.6 ± 174.2 [a]
**Decorin**	18263.7 ± 878.5 [a]	15549.4 ± 1047.7[a]	21100.4 ± 969.0 [a]	20700.6 ± 3002.2 [a]
**IFN-α**	557.4 ± 46.5 [b]	743.2 ± 67.7 [a]	333.4 ± 51.9 [c]	675.6 ± 79.7 [a,b]
**IFN-β**	327.6 ± 50.9 [b]	575.1 ± 75.5 [a]	171.3 ± 17.7 [c]	444.0 ± 127.1 [a,b]
**IGF-2**	793.0 ± 114.1 [a,b]	1103.0 ± 72.6 [a]	656.3 ± 80.3 [b]	1126.0 ± 172.2 [a]

**Anti-inflammatory mediators**				
**EPO**	319.8 ± 21.6	326.9 ± 54.7	292.5 ± 31.1	320.9 ± 30.0
**IL-4**	262.9 ± 19.8	345.8 ± 80.5	265.2 ± 17.1	357.1 ± 89.4
**IL-10**	611.3 ± 75.6 [a]	596.8.1 ± 67.8 [a]	408.2 ± 14.3 [b]	664.4 ± 166.1 [a]
**IL-1ra**	703.2 ± 38.7	684.4 ± 22.8	657.2 ± 16.7	602.6 ± 128.7
**Insulin**	345.0 ± 37.6 [a]	339.7 ± 49.6 [a]	217.3 ± 8.9 [b]	363.0 ± 67.3 [a]
**PDGF-BB**	551.0 ± 33.6 [a]	568.8 ± 52.8 [a]	435.3 ± 32.4 [b]	509.9 ± 148.9 [a]
**PECAM-1**	1138.9 ± 42.4 [a]	919.6 ± 123.5 [a]	784.4 ± 173.9 [b]	595.4 ± 74.6 [b]
**TGF-α**	517.4 ± 49.8 [a]	570.7 ± 31.4 [a]	164.7 ± 35.2 [b]	430.1 ± 83.2 [a]
**TGF-β1**	300.7 ± 49.2 [b]	455.1 ± 83.3 [a]	224.2 ± 21.8 [b]	396.5 ± 92.1 [b]
**TIMP-1**	749.7 ± 69.8 [b]	935.5 ± 64.2 [a]	455.4 ± 54.3 [b]	483.9 ± 145.0 [a]
**TIMP-2**	4079.4 ± 651.8 [a]	5521.6 ± 512.9 [a]	3559.5 ± 389.9 [a]	3396.7 ± 1087.7 [a]
**FGF-21**	335.2 ± 19.1 [a]	168.4 ± 52.8 [b]	270.4 ± 31.02 [a]	240.3 ± 28.3 [a]

*post-weaned piglets were fed for 21 days with a control diet (control and LPS groups) or SYN diet (SYN and SYN+LPS groups). At the end of the feeding trial, piglets from the LPS and SYN+LPS groups were challenged with 80µg/b.w. LPS. After 3h, colon samples from all animals were collected and analyzed for inflammatory markers expression by protein array (n=4).

** the results were expressed as arbitrary units (A.U.) and are presented as average ± standard error of the mean (mean ± SEM). The effects of experimental diets and of LPS challenge on the protein expression were analyzed using two-way Anova and Fisher tests. Values of $p < 0.05$ were considered significant.

[a,b,c] Mean values within a row with unlike superscript letters were significantly different ($p<0.05$).
